# Adult-onset type 1 diabetes: predictors of major cardiovascular events and mortality

**DOI:** 10.1093/eurheartj/ehaf304

**Published:** 2025-05-14

**Authors:** Yuxia Wei, Tomas Andersson, Tiinamaija Tuomi, Thomas Nyström, Sofia Carlsson

**Affiliations:** Institute of Environmental Medicine, Karolinska Institutet, Nobels väg 13, 17177 Stockholm, Sweden; Institute of Environmental Medicine, Karolinska Institutet, Nobels väg 13, 17177 Stockholm, Sweden; Department of Endocrinology, Abdominal Center, Helsinki University Hospital, Helsinki, Finland; Genetics Research Program, Folkhälsan Research Center, Helsinki, Finland; Institute for Molecular Medicine Finland (FIMM), University of Helsinki, Helsinki, Finland; Department of Clinical Sciences Malmö, Lund University, Skåne University Hospital, Malmö, Sweden; Department of Clinical Science and Education, Karolinska Institutet, Stockholm, Sweden; Institute of Environmental Medicine, Karolinska Institutet, Nobels väg 13, 17177 Stockholm, Sweden

**Keywords:** Adult-onset, Type 1 diabetes, Cardiovascular diseases, All-cause mortality, Cause-specific mortality, Prognostic factors

## Abstract

**Background and Aims:**

The prognosis of adult-onset type 1 diabetes (T1D) and prognostic factors are sparsely investigated. This study assessed mortality, major adverse cardiovascular events (MACE), and prognostic factors in adult-onset T1D, particularly focusing on those diagnosed at age ≥40.

**Methods:**

Participants were people diagnosed with adult-onset T1D (*n* = 10 184) or type 2 diabetes (T2D, n = 375 523) in 2001–20 from the Swedish National Diabetes Register and 509 172 population controls from the Total Population Register, followed until 2022. Hazard ratios (HR) and population attributable risk fraction (PAR%) were estimated.

**Results:**

People with T1D had higher incidence of MACE (HR 1.30 [95% confidence interval 1.17, 1.45]), all-cause mortality (1.71 [1.60, 1.84]), and mortality from cardiovascular or non-cardiovascular diseases, cancer, or infection than population controls. They had lower MACE incidence (0.67 [0.60, 0.75]) and higher mortality from diabetic coma or ketoacidosis (7.04 [4.54, 10.9]) than people with T2D. Smoking (PAR% 10.7%) and glycated haemoglobin (HbA1c) ≥ 53 mmol/mol (10.4%) accounted for most deaths while overweight/obesity (19.8%), smoking (8.4%), and high HbA1c (8.8%) accounted for most MACE events in T1D. Results were similar for T1D diagnosed at age ≥40, although they had lower insulin pump use and higher HbA1c than people diagnosed earlier.

**Conclusions:**

Adult-onset T1D carries excess risk of death and MACE compared with population controls but less MACE risk than T2D. Individuals diagnosed after age 40 had similar excess risk and poorer glycaemic control than those diagnosed earlier, underscoring the need for improved management. Key prognostic factors were smoking, poor glycaemic control, and overweight/obesity.

## Structured Graphical Abstract

**Structured Graphical Abstract:**
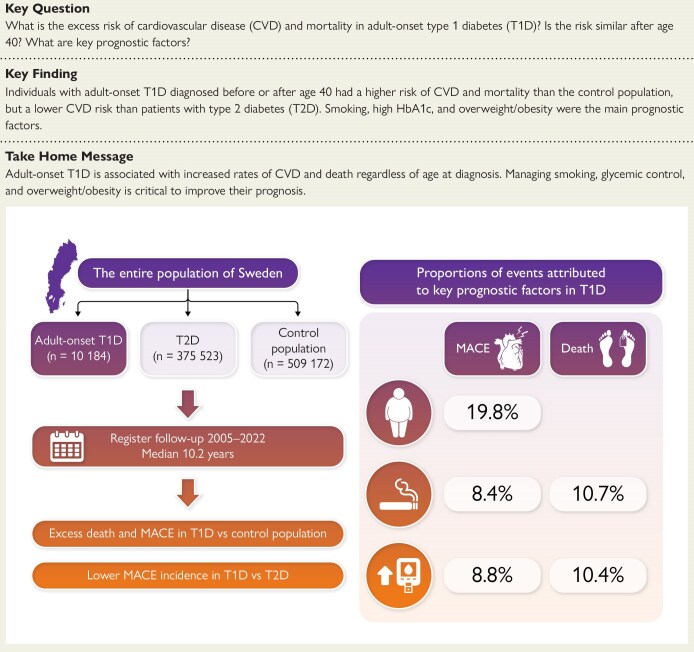

**See the editorial comment for this article ‘Adult-onset type 1 diabetes: new insights but still much to learn’, by D.J. Magliano and O. Rolandsson, https://doi.org/10.1093/eurheartj/ehaf427.**

## Introduction

People with type 1 diabetes (T1D) face an increased risk of mortality and cardiovascular diseases (CVD) despite major advances in diabetes management over the last decades.^1^ Thus, dedicated studies on mortality and CVD in T1D are crucial, as evidence is often extrapolated from research in type 2 diabetes (T2D) or the general population.^2^ Key risk factors contributing to poor prognosis include poor glycaemic control, high blood pressure, unfavourable lipid profiles, and smoking.^3–11^ Current knowledge on the prognosis of T1D primarily stems from studies of T1D diagnosed during childhood or adolescence whereas less is known regarding T1D diagnosed later in life. This is particularly evident for people diagnosed with T1D after the age of 40, although they represent a significant proportion of patients. In fact, the median age at T1D diagnosis was recently estimated to be 29 years.^12^ T1D shows substantial heterogeneity by onset-age, with people diagnosed during adulthood having higher residual β-cell function and less frequent ketoacidosis at diagnosis, and being less likely to have multiple autoantibodies than people diagnosed during childhood.^13,14^ Better residual insulin secretion and shorter diabetes duration at a given age imply less exposure to hyperglycaemia in T1D diagnosed at higher ages, which could result in a lower risk of mortality/CVD. In support hereof, a Swedish study investigating T1D diagnosed at age ≤30 years reported that the excess risk of CVD and mortality attenuated with age at diagnosis.^10^ Whether this applies to T1D diagnosed at higher ages is unknown.

Among the few studies investigating the prognosis of adult-onset T1D across all ages, studies from Sweden (*n* = 1573)^15^ and Korea (*n* = 8523)^16^ report higher mortality rates in T1D compared with T2D, which seems to contradict the hypothesis that T1D is milder at higher ages. Interestingly, the largest study to date, involving 35 355 patients in England and Wales, revealed that only men with T1D had higher mortality rates than those with T2D, highlighting a notable sex difference.^17^ However, the underlying causes of death contributing to the excess mortality in adult-onset T1D remain unknown. Previous findings on CVD are inconsistent, with the Korean study reporting higher CVD incidence in adult-onset T1D than in T2D^16^ whereas we recently observed a lower incidence of CVD in T1D than in T2D.^15^ Our study was however small (*n* = 1573) and further investigation on the prognosis of adult-onset T1D is needed. To our knowledge, no study has investigated the role of different prognostic factors in adult-onset T1D, and potential sex differences also remain to be elucidated.

We aimed to study the prognosis of adult-onset T1D, especially for those diagnosed at age ≥40 years, and elucidate risk factors contributing to poor prognosis. To this aim, we investigated all-cause mortality, cause-specific mortality, and incident CVD in adult-onset T1D, T2D and population controls, using nationwide Swedish data for the period 2001 to 2022.

## Methods

### Study population

We identified everyone with adult-onset (≥18 years) T1D and T2D (≥18 years) diagnosed 2001–20 from the National Diabetes Register (NDR).^18^ The date of diagnosis was defined as the first record of diabetes in NDR or the National Patient Register (NPR), or the first record of glucose-lowering drugs in the National Prescribed Drug Register (NPDR). Details about the exclusion criteria and different registers are provided in Supplementary data online, Method  *S1*. People were further categorized by age at diagnosis (18–29, 30–39, and ≥40 years). In the year of diagnosis, everyone with T1D was matched by age, sex, and county to 50 population controls free of diabetes from the Total Population Register, which records the entire population of Sweden. The final study population included 10 184 people with T1D, 375 523 with T2D, and 509 172 population controls. People with prevalent CVD at cohort entry were excluded from the analysis of CVD incidence. This study was approved by the Swedish Ethical Review Authority (2022–02549-02).

### Covariates and prognostic factors

Information on sex, date, and country of birth was obtained from the Total Population Register, while information on marital status and education was obtained from the longitudinal integration database for health insurance and labour market studies (LISA). NDR consecutively records information on smoking, body mass index (BMI), physical activity, glycated haemoglobin (HbA1c), blood pressure, serum lipid profile, and estimated glomerular filtration rate (eGFR), albuminuria, and insulin regimen since diabetes diagnosis (see Supplementary data online, Method  *S2*). We also calculated the estimated glucose disposal rate (eGDR) as a proxy for insulin resistance (see Supplementary data online, Method  *S2*).

### Outcomes

Mortality outcomes included all-cause mortality, cardiovascular death, non-cardiovascular death, death from cancer, infection, and diabetic coma or ketoacidosis. The causes of death Register provided information on date and causes of death (ICD codes in Supplementary data online, Method  *S3*). Major adverse cardiovascular events (MACE) was used as CVD outcome, defined as cardiovascular death, or the first inpatient record of nonfatal myocardial infarction or nonfatal stroke in NPR (see Supplementary data online, Method  *S3*).

### Statistical analysis

Baseline characteristics were presented as proportions for categorical variables and median (inter-quartile range) or mean (standard deviation) for continuous variables.

The analysis of mortality/MACE and prognostic factors involves: (i) comparing mortality/MACE incidence in T1D, population control, and T2D; (ii) identifying prognostic factors associated with mortality/MACE and calculating population attributable risk fractions (PAR%) in people with T1D; (iii) estimating the association of the number of risk factors with mortality/MACE in T1D, with population controls as the reference group; and (iv) describing trajectories of prognostic factors in T1D.

#### Mortality and MACE incidence in T1D, population controls, and T2D

People diagnosed with diabetes in 2001–2005 entered the cohort on 31 December 2005, since the insulin treatment information, used to ensure the correct classification of T1D, was available since 2005. People diagnosed 2006 onwards entered the cohort at date of diagnosis. Population controls entered the cohort on the same date as their matched T1D counterparts. Participants were followed until the date of the outcome occurrence, death, emigration, diabetes diagnosis (for controls), or end of follow-up (8 June 2022 for all-cause mortality, 30 June 2021 for cause-specific mortality and 31 December 2021 for MACE, due to availability of register information), whichever came first.

We plotted the cumulative probability of different outcomes in T1D and their matched controls over diabetes duration (duration since matching; Supplementary data online, Method  *S4*). We estimated hazard ratios (HRs) and 95% confidence intervals (CIs) for all-cause mortality, cause-specific mortality, and MACE in people with T1D vs. population controls and people with T2D, overall, and stratified by age at diagnosis/matching (18–29, 30–39, and ≥40 years). Cox models with comparison to population controls were stratified by matching groups, with follow-up duration as the time scale, and with adjustment for education, country of birth, and marital status (Model_vscontrol_). Models with comparison to T2D were fitted with attained age as the time scale to ensure optimal adjustment for age,^19^ and with adjustment for age and calendar year at diagnosis, sex, education, marital status, and country of birth (Model_indiabetes_). We also performed sex-specific analyses.

#### Association of individual prognostic factors with mortality and MACE in T1D

Within individuals with T1D, we estimated HRs of all-cause mortality and MACE for each of the following factors: smoking, physical activity, BMI, HbA1c, blood pressure, triglycerides, eGFR, and albuminuria, using the first measurement (median diabetes duration 1.5 years; Supplementary data online, Table  *S1*; Method  *S5*) since cohort entry. The main model for each factor was additionally adjusted for diabetes duration at lifestyle/biomarker measurement on the basis of Model_indiabetes_. To estimate the proportion of adverse events attributable to each risk factor, we calculated PAR% by including all prognostic factors in the same model (mutually adjusted model, Supplementary data online, Method  *S5*). Missing data were imputed using the multi-variate imputation by chain equations (MICE; Supplementary data online, Method  *S6*). To ensure reliability of the imputation, we checked variable distributions before and after imputation (see Supplementary data online, Figure  *S1*) and performed complete-case analyses as sensitivity analyses.

#### Mortality and MACE incidence by number of risk factors

Factors associated with mortality (or MACE) were used to categorize people with T1D by number of risk factors (0, 1, 2, or ≥3). We then estimated the HRs of different outcomes in different categories of people with T1D, with their matched population controls as the reference group. Models were fitted in the same way as Model_vscontrol_.

#### Trajectories of prognostic factors in T1D

We estimated the trajectories of different prognostic factors over diabetes duration in people with T1D by age at diagnosis (18–29, 30–39, and ≥40 years) using generalized linear models^20^ (see Supplementary data online, Method  *S7*).

Statistical analyses were performed using R 4.3.1 and Stata 17.0. All tests were 2-sided, with *P* < .05 indicating statistical significance.

## Results

### Baseline characteristics

Among the 10 184 individuals with T1D, 40.4% (*n* = 4113) were diagnosed at age 18–29 years, 21.3% (*n* = 2172) at age 30–39 years, and 38.3% (*n* = 3899) at age ≥40 years (40–102). People aged ≥40 years were more likely to be female and smokers, had higher blood pressure, triglycerides, and Hb1Ac, and worse kidney function, and more severe insulin resistance (lower eGDR) than those diagnosed younger. They also had higher BMI and were less likely to experience diabetic coma or ketoacidosis than those diagnosed at age 18–29 years (*Table 1*). We observed no major difference in clinical biomarkers including HbA1c between men and women with T1D (see Supplementary data online, Table  *S2*). Compared with controls, a higher proportion of those with T1D were born in Sweden and the proportion with high education was slightly lower. People with T1D had higher HbA1c, but lower prevalence of smoking, physical inactivity, and albuminuria, and lower BMI, blood pressure, and triglycerides than people diagnosed with T2D at similar ages (see Supplementary data online, Table  *S3*).

**Table 1:** Baseline characteristics of people with type 1 diabetes by age at diagnosis and population controls

Baseline characteristics	Population controls by age at matching			People with T1D by age at diagnosis				
	18–29 years	30–39 years	≥40 years	18–29 years	30–39 years	≥40 years	*P* value^a^	*P* value^b^
*N*	205 649	108 600	194 923	4113	2172	3899		
Men, %	62.1	62.2	53.9	62.1	62.2	53.9	.9066	<.0001
Born in Sweden,%	91.2	87.0	87.7	95.6	92.0	91.3	<.0001	<.0001
Married, %	3.4	21.8	51.8	5.1	33.5	50.7	<.0001	<.0001
Post-secondary or higher education, %	40.0	45.7	34.2	40.6	42.3	32.4	.2096	<.0001
Smoking^c^, %				12.8	15.6	17.8	.0754	.0003
Physically inactive^c^, %				4.6	6.5	6.1	.0898	.1021
BMI^c^, mean (SD)				23.4 (4.3)	24.7 (4.5)	25.2 (4.3)	<.0001	<.0001
HbA1c^c^, median (IQR)				66.0 (54.0, 84.0)	68.0 (53.0, 85.0)	72.0 (57.0, 89.0)	.6463	.0001
Systolic blood pressure^c^, median (IQR)				118 (110, 125)	120 (110, 128)	127 (119, 138)	.0001	.0001
Triglycerides^c^, median (IQR)				0.9 (0.7, 1.3)	1.0 (0.7, 1.6)	1.2 (0.9, 1.8)	.0005	.0001
eGFR^c^, median (IQR)				119 (104, 139)	109 (95, 127)	96 (83, 113)	.0001	.0001
Albuminuria^c^, %				2.0	3.1	8.1	.1593	<.0001
Diabetic coma or ketoacidosis^c^, %				14.7	8.1	8.4	<.0001	<.0001
eGDR^d^				8.65	8.33	7.08	<.0001	<.0001

T1D, type 1 diabetes; BMI, body mass index; SD, standard deviation; HbA1c: glycated hemoglobin; IQR, inter-quartile range; eGFR, estimated glomerular filtration rate; eGDR, estimated glucose disposal rate, a proxy for insulin resistance.

^a^ *P* value for comparison between people with T1D diagnosed at age 30–39 years and at age 18–29 years.

^b^ *P* value for comparison between people with T1D diagnosed at ages ≥40 years and at age 18–29 years.

^c^Measured within 3 months of diabetes diagnosis.

^d^Estimated with adjustment for diabetes duration, country of birth, education, marital status, and sex.

### Mortality and MACE incidence in T1D, population controls, and T2D

During a median follow-up of 10.2 years, there were 816 deaths (202 from cardiovascular causes), and 361 MACE events in people with T1D (see Supplementary data online, Table  *S4*). We also recorded 26 888 deaths and 14 034 MACE events in controls and 90 921 deaths and 48 374 MACE events in people with T2D. The leading cause of death in T1D was diabetic coma or ketoacidosis in people diagnosed at age <40 years, and CVD and cancer in those diagnosed at age ≥40 years (see Supplementary data online, Table  *S4*). In population controls and patients with T2D, CVD and cancer were the leading causes of death across all age groups.

**Figure 1:**
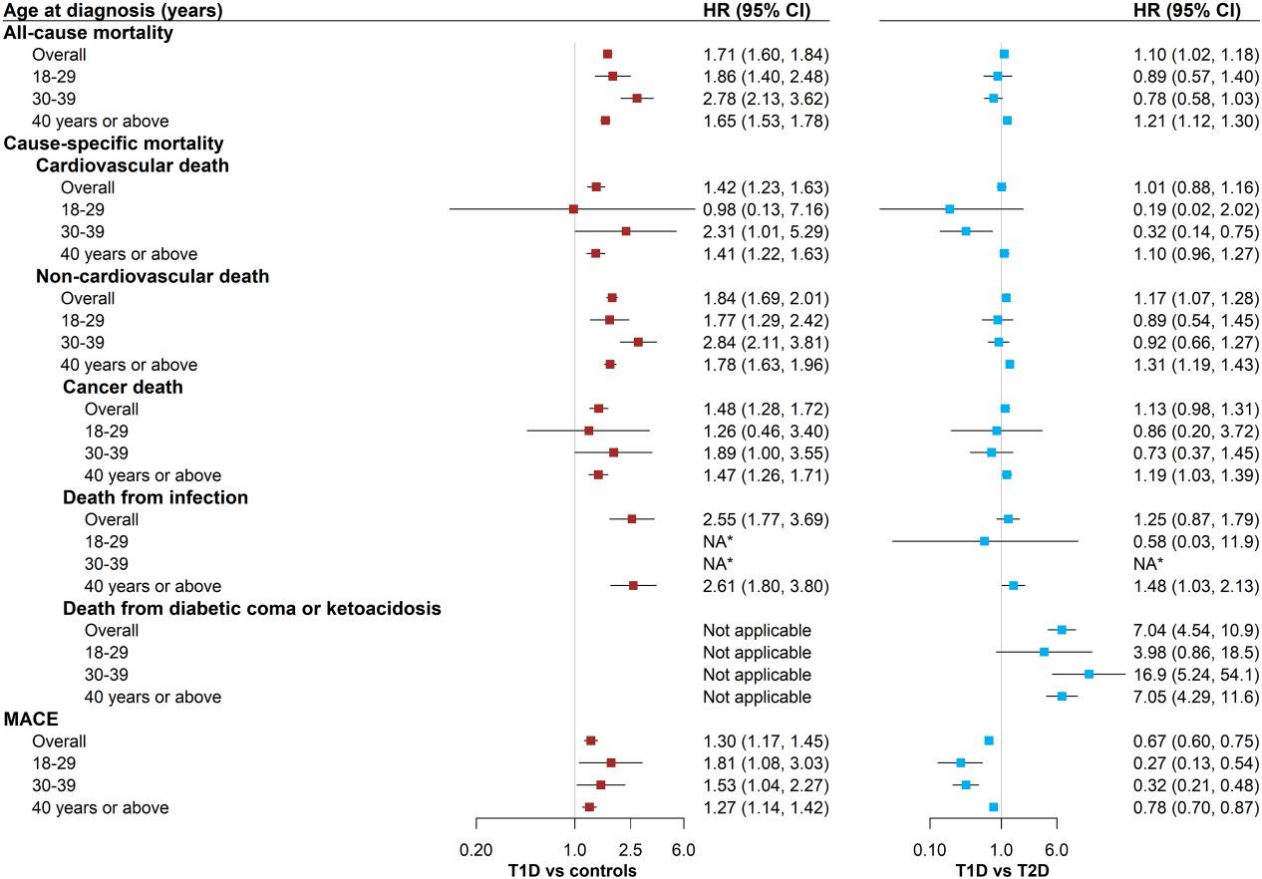
Hazard ratios (95% CI) for mortality and MACE in adult-onset T1D compared with population controls and people with T2D by age at diagnosis/matching. HR, hazard ratio; CI, confidence interval; MACE, major adverse cardiovascular events; T1D, type 1 diabetes; T2D, type 2 diabetes. * The number of events was too low to estimate HR (95% CI)

All-cause mortality (HR 1.71; 95% CI 1.60, 1.84) and most kinds of cause-specific mortality including cardiovascular death, and non-cardiovascular death such as death from cancer or infection was higher in T1D than controls (*Figure 1*; Supplementary data online, Figure  *S2*). They also had a higher incidence of MACE (HR 1.30; 95% CI 1.17, 1.45; *Figure 1*). The elevated risks were observed across the three age groups. There was no attenuation in HRs with increasing age (*Figure 1*) while the largest absolute risk difference was observed in the oldest group (see Supplementary data online, Figure  *S3*). Overall and in those aged ≥40 years, the excess risk of all-cause mortality, non-cardiovascular death, cancer death, and death from infection in T1D (vs. controls) was more pronounced in men than in women while the opposite pattern was seen for MACE (see Supplementary data online, Figures  *S4*–*S5*).

The risk of death from diabetic coma or ketoacidosis in T1D was seven times as high as in people with T2D (HR 7.04, 95% CI 4.54, 10.9), and the excess risk was observed across all age groups (*Figure 1*) and in both men and women (see Supplementary data online, Figure  *S6*); T1D diagnosed at age ≥40 years also had slightly higher rates of all-cause mortality, non-cardiovascular death, cancer death, and death from infection than T2D (*Figure 1*) and the excess risk was limited to men (see Supplementary data online, Figure  *S6*). In contrast, the incidence of cardiovascular deaths was similar between T1D and T2D while people with T2D had a higher incidence of MACE across all the three age groups (*Figure 1*).

### Association of individual prognostic factors with mortality and MACE in T1D

In people with T1D, smoking, being underweight or obese, physical inactivity, as well as having HbA1c, blood pressure, triglycerides, eGFR out of target, and albuminuria were associated with increased all-cause mortality (*Table 2*). The eight risk factors were confirmed in the complete-case analysis (see Supplementary data online, Table  *S5*). Similar results were found for MACE incidence, although 95% CIs for risk estimates concerning physical activity and being underweight cross 1 (*Table 2*). Calculation of PAR% indicated that HbA1c above target (10.4% overall vs. 6.8% in those diagnosed at age ≥40 years) and smoking (10.7% vs. 12.0%) accounted for most deaths in people with T1D overall and in those diagnosed at age ≥40 years (*Table 2*). Overweight/obesity (19.8% vs. 20.8%), HbA1c out of target (8.8% vs. 7.3%), and smoking (8.4% vs. 8.9%) accounted for the most MACE events in T1D, overall and in T1D diagnosed at age ≥40 years (*Table 2*).

**Table 2:** Hazard ratios for all-cause mortality and MACE in adult-onset type 1 diabetes by baseline prognostic factors

Lifestyle and clinical characteristics	Overall				T1D diagnosed at age ≥40 years			
	Proportion, %	Main model	Mutually adjusted model	PAR%	Proportion, %	Main model	Mutually adjusted model	PAR%
All-cause mortality								
Smoking vs. non-smoking	15.6	1.81 (1.50, 2.18)	1.76 (1.41, 2.19)	10.7	19.0	1.73 (1.43, 2.10)	1.70 (1.35, 2.14)	12.0
Being physically inactive vs. active	5.8	1.55 (1.22, 1.97)	1.38 (1.10, 1.74)	2.6	6.1	1.63 (1.27, 2.11)	1.46 (1.15, 1.86)	3.3
BMI								
Underweight	2.6	1.90 (1.16, 3.11)	2.03 (1.25, 3.29)	2.7	1.9	1.75 (0.99, 3.07)	1.90 (1.08, 3.33)	1.5
Normal weight	55.5	1.00	1.00		49.4	1.00	1.00	
Overweight	31.4	1.04 (0.85, 1.26)	0.98 (0.79, 1.20)	−	35.6	1.13 (0.91, 1.41)	1.08 (0.85, 1.36)	−
Obesity	10.4	1.41 (1.10, 1.79)	1.22 (0.92, 1.62)	2.5	13.0	1.48 (1.13, 1.95)	1.31 (0.97, 1.77)	4.2
HbA1c out of target	49.8	1.35 (1.15, 1.58)	1.23 (1.05, 1.43)	10.4	53.0	1.23 (1.04, 1.45)	1.12 (0.95, 1.32)	6.8
Blood pressure out of target	39.8	1.21 (1.02, 1.44)	1.14 (0.96, 1.36)	6.1	54.1	1.13 (0.93, 1.37)	1.06 (0.88, 1.28)	4.2
Triglycerides out of target	13.8	1.39 (1.13, 1.70)	1.15 (0.90, 1.45)	2.0	18.9	1.37 (1.10, 1.70)	1.11 (0.87, 1.42)	2.1
eGFR out of target	2.6	1.31 (1.05, 1.65)	1.29 (1.01, 1.64)	0.9	6.4	1.28 (1.02, 1.61)	1.25 (0.98, 1.60)	1.7
Albuminuria	5.6	1.62 (1.32, 1.99)	1.46 (1.20, 1.77)	2.8	9.6	1.63 (1.32, 2.00)	1.47 (1.21, 1.79)	1.9
MACE								
Smoking vs. non-smoking	15.4	1.60 (1.12, 2.28)	1.62 (1.12, 2.32)	8.4	18.8	1.50 (1.01, 2.23)	1.52 (1.01, 2.28)	8.9
Being physically inactive vs. active	5.6	1.42 (0.88, 2.29)	1.24 (0.76, 2.01)	1.2	5.5	1.54 (0.99, 2.39)	1.34 (0.86, 2.08)	1.8
BMI								
Underweight	2.6	1.71 (0.73, 3.99)	1.86 (0.80, 4.33)	2.3	1.9	1.53 (0.63, 3.72)	1.71 (0.70, 4.17)	1.3
Overweight	31.3	1.56 (1.16, 2.10)	1.47 (1.06, 2.03)	12.1	35.4	1.50 (1.10, 2.05)	1.41 (0.99, 1.99)	12.2
Obesity	10.2	2.15 (1.50, 3.09)	1.82 (1.18, 2.82)	7.7	12.5	2.09 (1.44, 3.04)	1.76 (1.10, 2.81)	8.6
HbA1c out of target	49.5	1.33 (1.04, 1.69)	1.19 (0.91, 1.54)	8.8	52.4	1.26 (0.98, 1.63)	1.13 (0.86, 1.49)	7.3
Blood pressure out of target	39.4	1.30 (1.01, 1.68)	1.16 (0.89, 1.51)	5.6	54.1	1.20 (0.91, 1.58)	1.07 (0.81, 1.42)	4.0
Triglycerides out of target	13.5	1.72 (1.26, 2.35)	1.31 (0.91, 1.90)	4.2	18.3	1.73 (1.20, 2.49)	1.33 (0.86, 2.06)	5.7
eGFR out of target	2.2	1.46 (1.00, 2.14)	1.39 (0.95, 2.02)	0.7	5.3	1.46 (1.00, 2.15)	1.39 (0.95, 2.03)	1.8
Albuminuria	5.2	1.40 (0.98, 2.00)	1.17 (0.83, 1.65)	0.9	8.8	1.43 (0.99, 2.05)	1.20 (0.85, 1.71)	0.7

The basic models were fitted with attained age as the time scale, with adjustment for age and year at T1D diagnosis, diabetes duration at NDR visit, country of birth, education, marital status, and with stratification by sex. The full models were additionally adjusted for all variables in the table.

T1D, type 1 diabetes; BMI, body mass index; HbA1c: glycated hemoglobin; eGFR, estimated glomerular filtration rate; MACE, major adverse cardiovascular event; PAR%, population attributable risk fraction.

### Mortality and MACE incidence by number of risk factors

**Figure 2:**
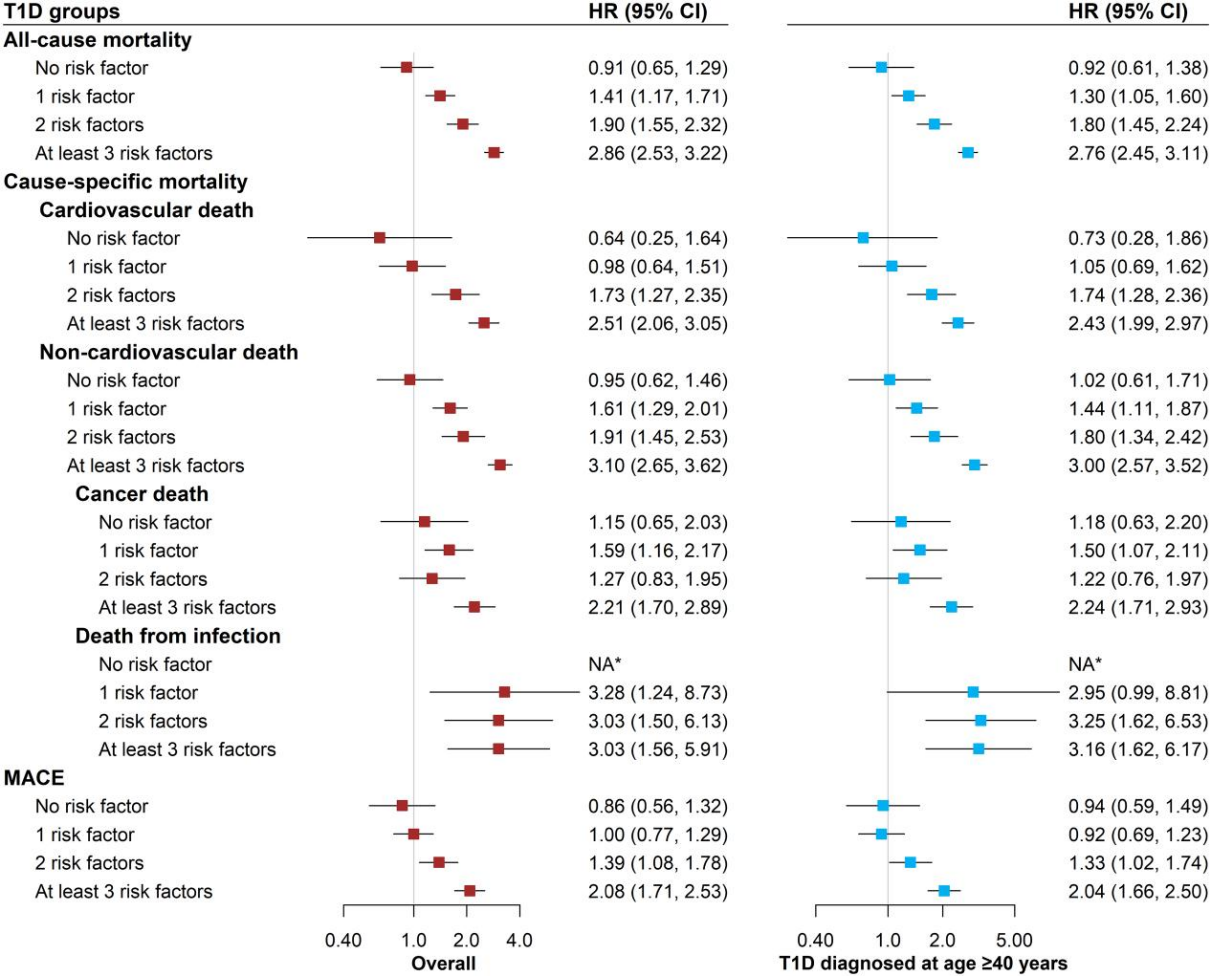
Hazard ratios for mortality and MACE in people with T1D compared with matched population controls by number of risk factors. T1D, type 1 diabetes; HR, hazard ratio; CI, confidence interval; MACE, major adverse coronary events. Risk factors: smoking, physical inactivity, underweight/obesity, albuminuria, and HbA1c, blood pressure, triglycerides, and eGFR out of target. * The number of events was too low to estimate HR (95% CI)

There was a dose-response relationship between the number of risk factors and excess risk of all-cause mortality, cardiovascular death, non-cardiovascular death (including death from cancer and infection) and MACE observed in people with T1D vs. controls (*Figure 2*; Supplementary data online, Table  *S6*). For example, the HR (95% CI) of all-cause mortality was 1.41 (1.17, 1.71), 1.90 (1.55, 2.32), and 2.86 (2.53, 3.22) in those with 1, 2, or at least 3 risk factors, respectively. These results were similar in the subset diagnosed with T1D at age ≥40 years (*Figure 2*). People with T1D without any of the eight risk factors did not have a higher incidence of mortality or MACE than population controls.

### Trajectories of prognostic factors in T1D

**Figure 3:**
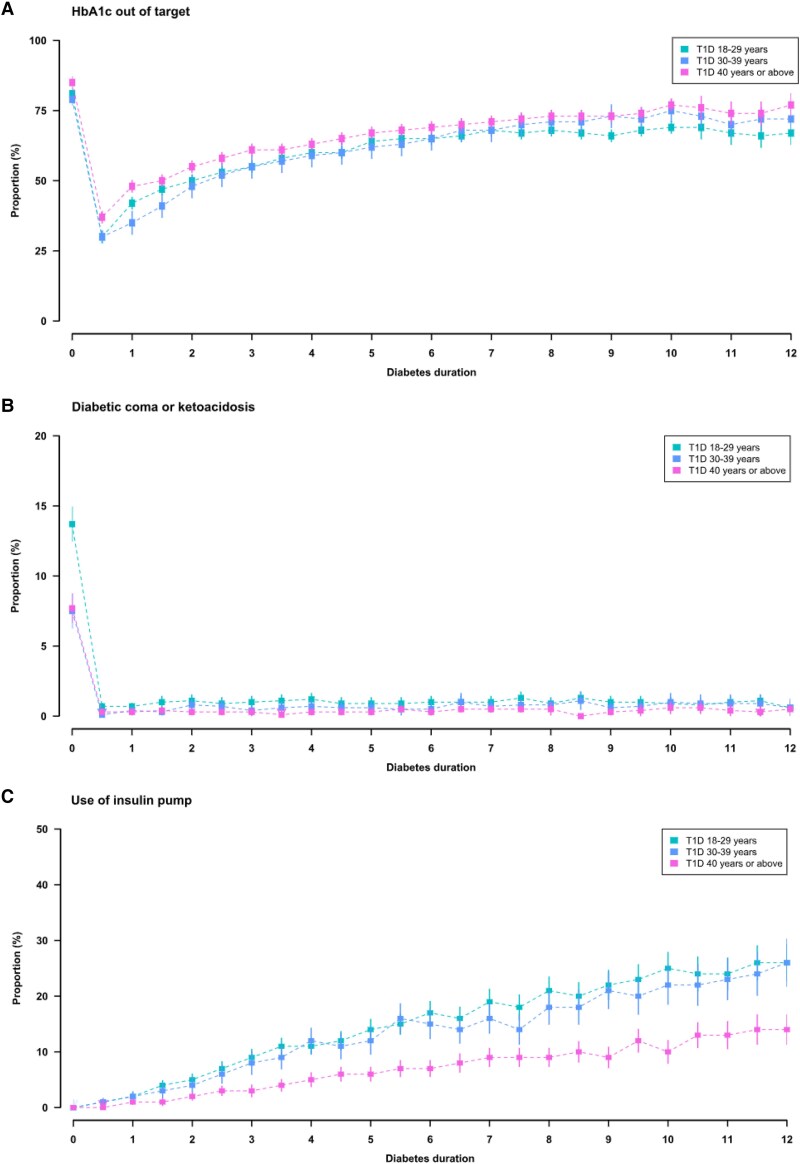
Trajectories of HbA1c (A), severe glycaemic events (B), and insulin regimens (C) over diabetes duration in adult-onset T1D. HbA1c, glycated haemoglobin; T1D, type 1 diabetes; HbA1c out of target, HbA1c ≥ 7% (53 mmol/mol)

Most people with T1D were initially treated with insulin injections but the use of insulin pumps increased with diabetes duration. The proportion using pumps was lower in people diagnosed at age ≥40 than in those diagnosed earlier, e.g. at the end of follow-up, 14% of the older age group was using pumps compared with 26% of younger patients (*Figure 3*). Men and people with lower education were less likely to use insulin pumps (see Supplementary data online, Table  *S2*, *Table S7*). People diagnosed at age ≥40 had worse glycaemic control than those diagnosed at younger ages. After 12 years from diagnosis, 77% and 67% of people diagnosed at age ≥40 and 18–29 years did not reach the HbA1c target, respectively (*Figure 3*). The occurrence of severe glycaemic events (coma or ketoacidosis) was highest in those diagnosed at age 18–29 years. People diagnosed at age ≥40 years had a higher prevalence of smoking and albuminuria, and worse levels of blood pressure, lipids, and eGFR than those diagnosed at younger ages (see Supplementary data online, Figure  *S7*).

## Discussion

In this Swedish nationwide population-based study, we found that the risk of all-cause mortality, almost all types of cause-specific mortality, and MACE was 50% to 2-fold higher in adult-onset T1D than in population controls, and the risk of death from diabetic coma or ketoacidosis was seven times as high as in T2D, regardless of age at diagnosis (*Structured Graphical Abstract*). Our findings imply that adult-onset T1D is characterized by a dual burden of acute and chronic complications. People diagnosed with T1D at age ≥40 years were more insulin resistant, had worse glycaemic control, were more likely to smoke, and less likely to use insulin pumps compared with those diagnosed at a younger age. We also note that T1D diagnosed at age ≥40 years does not have better long-term prognosis than adult-onset T1D diagnosed at younger ages and identify a need for improved management. In addition, we found that smoking, BMI, and HbA1c are the key contributors to adverse outcomes, accounting for ∼40% of cardiovascular events and 20% of deaths in people with adult-onset T1D.

### Mortality

Previous studies, including our much smaller study, also showed higher mortality in adult-onset T1D than in population controls.^15,16^ We extend these findings by showing, for the first time, that people with adult-onset T1D have excess risk of death not only from CVD and infection, which is to be expected, but also from cancer. We also noted a much higher incidence of death from diabetic coma and ketoacidosis in T1D than in T2D, which was seen across all ages and both sexes. An important observation was that the excess mortality did not decrease with age at diagnosis, in contrast to previous observations in T1D diagnosed at age ≤30.^10^ In fact, the absolute risk difference in mortality and MACE between people with T1D and controls was greater for those diagnosed after age 40. This indicates that T1D is as severe a condition whether diagnosed during young adulthood or middle age and those diagnosed at higher ages need as careful monitoring as those diagnosed younger.

Consistent with an UK study, all-cause mortality rates in T1D were higher than in T2D, but only in men.^17^ Additionally, men with T1D diagnosed at age ≥40 years had a higher risk of death from diabetic coma or ketoacidosis, cancer, and infection than men with T2D. The reason behind this sex difference is unclear. In our population, men with T1D had higher prevalence of smoking and physical inactivity but did not have worse glycaemic control than women with T1D, and men and women did not differ in BMI, lipids, or eGFR levels. Similarly, the UK study observed male predominance in T1D mortality after adjusting for socioeconomic status, BMI, smoking, HbA1c, blood pressure, cholesterol, and eGFR. However, we observed significantly less insulin pump use in men (vs. women) with T1D, which may have contributed to their worse prognosis.

### MACE

The incidence of MACE in adult-onset T1D was higher than in population controls and the excess risk was higher in women than in men. This suggests that the female protection against CVD has been partly counteracted by diabetes, a phenomenon increasingly recognized in T2D^21^ but less reported in T1D. Compared with people with T2D, those with T1D had a lower incidence of MACE, confirming our previous findings,^15^ whereas the opposite was observed in a Korean study.^16^ This difference could reflect the differential improvement in diabetes care for T1D vs. T2D in Sweden. From 1998 to 2014, Sweden saw a 40% greater reduction in CVD events in T1D and a 20% greater reduction in T2D compared with population controls.^1^ It should also be noted that people with T1D had fewer cardiovascular risk factors including smoking, obesity, high blood pressure, and dyslipidaemia than those with T2D. Our findings highlight the role of insulin resistance and hyperinsulinemia—both of which are more pronounced in T2D than in T1D—in the development of vascular diseases.

### Prognostic factors

To our knowledge, this is the first study to investigate factors contributing to poor prognosis in adult-onset T1D. Overall and in those diagnosed after age 40 years, factors worsening T1D prognosis included smoking, physical inactivity, being underweight/overweight/obese, poor glycaemic control, high blood pressure, dyslipidemia, and poor kidney function, with a dose-response relationship between number of risk factors and MACE/mortality risk. The same factors are linked to adverse outcomes in childhood-onset T1D,^3,5,7,9,22–25^ although the relative importance of these factors in childhood-onset T1D^3^ differ from that in our study population. HbA1c out of target is one of the factors that contributed to the most adverse events, partly due to its high prevalence in this population. A large proportion of people with T1D did not reach the HbA1c target, particularly among those diagnosed at age ≥40 years (50%–80%). The worse glycaemic control at higher ages may be due to less frequent use of insulin pumps, which is linked to better glucose management than multiple dose injections.^26^ Our findings highlight the need for better glucose management in adult-onset T1D, including equal access and better adherence to advanced technology such as insulin pumps in older adults diagnosed with T1D,^27^ especially in men and people with low education. In addition, being overweight/obese accounted for the most MACE events, indicating the importance of weight management in people with T1D, especially those diagnosed after age 40 years, who had higher BMI and insulin resistance than people diagnosed younger.

### Strengths and limitations

We investigated a nationwide sample, with links to high-quality nationwide registers with virtually no loss to follow-up. This gave us access to detailed information on prognostic factors including lifestyle factors and biomarkers for almost everyone diagnosed with adult-onset T1D in Sweden. Unfortunately, no such data were available for controls. Some people with T1D had missing data on prognostic factors. This was handled by imputation^3,4^ and our complete-case analysis supported the validity of this approach. Regarding glycaemic control, we had access to HbA1c, which reflects the mean glucose level, but there was no information on glycaemic variability, which may also affect T1D prognosis.^28^ A T1D diagnosis before age 30 in NDR has a positive predictive value of 97%.^18^ There may be concerns about the validity of T1D diagnosed at higher ages. We tried to minimize misclassification by excluding T1D cases ever recorded with a contradictory diabetes type and those without insulin prescription. Only those alive at the end of 2005 were included, since treatment data used to ensure correct classification of T1D was available since 2005. This might lead to the underestimation of the excess mortality in people with diabetes, as those who were diagnosed in 2001–2005 and died before 2006 may be those with the worst prognosis. Importantly, this study was performed in people mostly of European ancestry and the generalizability of our findings to other countries and ethnicities is unclear. PAR% in particular is population-specific and different sets of risk factors may contribute to poor T1D prognosis in other settings.

## Conclusions

In conclusion, we found that people with adult-onset T1D had higher risk of all-cause mortality, mortality from both acute and chronic diseases, and CVD than diabetes-free individuals but had lower CVD incidence than individuals with T2D. People with T1D diagnosed at age ≥40 years did not have a better long-term prognosis than those diagnosed at younger ages. Poor glycaemic control, smoking, and overweight/obesity were the key factors contributing to poor prognosis. The dose-response relationship between the number of risk factors and adverse outcomes highlights the importance of intervening on as many risk factors as possible.

## Supplementary Material

ehaf304_Supplementary_Data

## Declarations

### Data Availability

Our data are register-based data that are pseudonymized and thus subject to GDPR and cannot be shared openly. Only metadata is published openly. Underlying data from the registers can be made available upon request to rdo@ki.se after ensuring compliance with relevant legislation and GDPR.

### Pre-registered Clinical Trial Number

The pre-registered clinical trial number is NCT06563401.
