# Supplementary material for: Adult-onset type 1 diabetes: predictors of major cardiovascular events and mortality
Source: Eur Heart J. 2025 May 14;46(38):3776–86. doi: 10.1093/eurheartj/ehaf304 (PMC12500326; doi:10.1093/eurheartj/ehaf304)
Supplement: ehaf304_Supplementary_Data [file ehaf304_supplementary_data.docx]

**Table of contents**

eMethods

Method S1. Exclusion criteria and different registers

Method S2. Assessment of covariates

Method S3. Assessment of outcomes

Method S4. Curves for cumulative probability of different outcomes

Method S5. Lifestyle/clinical characteristics affecting prognosis in people with type 1 diabetes (T1D)

Method S6. Imputation for lifestyles and clinical characteristics

Method S7. Trajectory analysis

Table S1. Diabetes duration at the time of measurement of prognostic factors

Table S2. Characteristics of people with type 1 diabetes by sex

Table S3. Baseline characteristics of people with type 2 diabetes according to age at diagnosis

Table S4. Number of participants and events for different outcomes according to diabetes status and age at diabetes diagnosis/matching

Table S5. Complete-case analysis for the association between prognostic factors and all-cause mortality in adult-onset type 1 diabetes

Table S6. Number of participants and events for different outcomes in population controls and people with T1D according to number of risk factors

Table S7. Distribution of prognostic factors in people with T1D according to age at diagnosis and educational levels

Figure S1. Distribution of variables in the original dataset and the five datasets with multivariate imputation by chained equations

Figure S2. Cumulative probability of different outcomes in T1D as compared to population controls over diabetes/matching duration

Figure S3. Cumulative probability of different outcomes in T1D by onset-age as compared to population controls over diabetes/matching duration

Figure S4. Cumulative probability of MACE in T1D diagnosed after age 40 years and matched controls by sex

Figure S5. Hazard ratios (95% CI) for mortality and MACE in adult-onset type 1 diabetes compared to population controls by age at diagnosis/matching and sex

Figure S6. Hazard ratios (95% CI) for mortality and MACE in adult-onset type 1 diabetes compared to type 2 diabetes by age at diagnosis and sex

Figure S7. Trajectories of lifestyle and clinical characteristics in people with T1D by onset-age

### **Method S1. Exclusion criteria and different registers**

We identified all people with adult-onset (≥18 years) T1D diagnosed in 2001-2020 from the National Diabetes Register (NDR), which was launched in 1996 and includes people with diabetes reported annually from primary care and specialist clinics in Sweden.^1^ Among those, we excluded everyone with a conflicting type of diabetes (primary diagnosis) recorded in the National Patient Register (NPR)^2^ at any point in 2001-2021, and anyone without a prescription of insulin recorded in the National Prescribed Drug Register (NPDR)^3^ in 2005-2021. NPDR includes all prescribed drugs dispensed at Swedish pharmacies since 2005.^3^ For comparison, we also included all individuals diagnosed with T2D at age ≥18 years in 2001-2020 recorded in NDR and without conflicting types of diabetes (primary diagnosis) recorded in NPR at any point in 2001-2021. To avoid diabetes secondary to pancreatic cancer, we excluded people diagnosed with cancer in the digestive system before or within 1 year after diabetes diagnosis and with pancreatic cancer as the underlying cause of death. In the year of diagnosis, everyone with T1D was matched by age, sex, and county to 50 population controls free of diabetes from the Total Population Register, which records the entire population of Sweden.

### **Method S2. Assessment of covariates**

The National Diabetes Register consecutively records information on smoking (yes or no: a smoker is a person who is a current smoker or has stopped smoking during the last 3 months),^4^ body weight and height, physical activity (reported using a five-graded scale from daily to never),^4^ glycated hemoglobin (HbA1c), blood pressure, serum lipid profile and estimated glomerular filtration rate (eGFR), albuminuria (microalbuminuria or macroalbuminuria), and insulin regimen (insulin pump or multiple daily injections)^5^ since diabetes diagnosis. Physical inactivity was defined as being active (corresponding to walking) for less than 30 minutes per week. We also calculated the estimated glucose disposal rate (eGDR) as a proxy for insulin resistance using the following formula: eGDR=19.02 - (0.22×BMI [kg/m^2^]) - (3.26 × hypertension [yes or no]) - (0.61×HbA1c [DCCT%]).^6^

### **Method S3. Assessment of outcomes**

Mortality outcomes included all-cause mortality, cardiovascular death, non-cardiovascular death, death from cancer, death from infection, and death from diabetic coma or ketoacidosis. We used major adverse cardiovascular events (MACE) as our CVD outcome. The Causes-of-Death Register provided information on date and causes of death (ICD-10 codes: I00-I99 for cardiovascular death, all codes except I00-I99 for non-cardiovascular death, C00-C97 for cancer death, A00-A99, B00-B99, U07.1, U07.2, U09.9, and U10.9 for death from infection, and E10.0, E10.1, E11.0, E11.1, E12.0, E12.1, E13.0, E13.1, E14.0, andE14.1 for death from diabetic coma or ketoacidosis). MACE was defined as cardiovascular death recorded in Causes-of-Death Register, or the first inpatient record (primary diagnosis and up to 7 contributory diagnoses)^7^ of nonfatal myocardial infarction (ICD-10: I21) or nonfatal stroke (ICD-10: I60-I64)^8-11^ in NPR.

### **Method S4. Curves for cumulative probability of different outcomes**

We used Kaplan-Meier curves to plot the cumulative probability of all-cause mortality in people with type 1 diabetes (T1D) and their matched controls over diabetes duration (duration since matching for controls). The curves for other outcomes were plotted using the cumulative incidence function from the Aalen-Johansen method (“plotCIF” function in the R package “Epi”) to account for competing events.

**Method S5 Lifestyle/clinical characteristics affecting prognosis in people with type 1 diabetes (T1D)**

To explore how and to what extent different factors affect the prognosis in people with type 1 diabetes (T1D), we first explored factors associated with all-cause mortality and major adverse cardiovascular events (MACE) in people with T1D. Cause-specific mortality was not investigated in this step due to the limit of statistical power. Potential prognostic factors investigated included smoking (yes vs no), physical inactivity (<1 time/week vs ≥1 time/week), BMI (underweight, overweight, or obesity vs normal weight), glycated hemoglobin (HbA1c) out of target (yes vs no), blood pressure out of target (yes vs no), triglycerides out of target (yes vs no), estimated glomerular filtration rate (eGFR) out of target (yes vs no), and albuminuria (yes vs no). Insulin regimens were not investigated since only a few people used insulin pumps at diabetes diagnosis. For potential prognostic factors, the first record in the National Diabetes Register (NDR) since cohort entry was used. If this measurement was within 1 year of diabetes diagnosis, the first measurement after 1 year of diagnosis was used instead. Follow-up duration for the analysis was therefore calculated from the date of the lifestyle/biomarker measurement to the date of the occurrence of outcomes, death, emigration, or 2022-06-08 for all-cause mortality (2021-12-31 for MACE, depending on the availability of different registers), whichever came first. The main models were fitted with attained age as the time scale and with adjustment for age and calendar year at diagnosis, sex, education, marital status, country of birth, and diabetes duration at the time of the NDR record. We further performed a mutual adjustment model including all lifestyle factors and clinical biomarkers to estimate the proportion of outcome events in the population that are attributable to different factors (population attributable risk fraction, PAR%). PAR% was calculated as (HR-1)*p/[(HR-1)*p+1]*100% where p is the proportion of the population with the risk factor and HR is the hazard ratio of the outcome in relation to the risk factor.^12^

We used factors identified to be associated with all-cause mortality in the main models to categorize people with T1D according to the number of risk factors (no risk factor, 1 risk factor, 2 risk factors, or at least 3 risk factors). We estimated the HRs of all-cause and cause-specific mortality in different categories, with their matched population controls as the reference group. Cox models were stratified by matching groups, with follow-up duration as the time scale, and with adjustment for education, country of birth, and marital status (Model_vscontrol_). Similarly, we categorized people with T1D according to the number of risk factors associated with MACE and estimated the HR of MACE in different categories as compared to population controls.

**Method S6 Imputation for lifestyles and clinical characteristics**

When exploring the associations of lifestyle/clinical factors with different outcomes, missing data for these factors in people with type 1 diabetes (T1D) were imputed using the multi-variate imputation by chain equations (MICE). The MICE algorithm assumes that missing only depends on observed values.^13^ Variables used in the imputation models included age and calendar year at T1D diagnosis, sex, educational level, marital status, county of residence, country of birth, diabetes duration, smoking, physical activity, body weight, standing height, HbA1c, systolic blood pressure, diastolic blood pressure, triglycerides, HDL cholesterol, LDL cholesterol, use of anti-hypertensive drugs, use of lipid-modifying agents, albuminuria, estimated glomerular filtration rate (eGFR), and prevalent ischemic heart disease, stroke, or heart failure. Five datasets with imputation were created using MICE, with 10 iterations for each dataset. When analyzing the associations with different outcomes, the results were pooled across the five datasets. The distributions of variables in the five complete datasets with imputation were similar with the distributions in the original dataset (**eFigure 1**), indicating the reliability of the imputation models. To further ensure the validity of the imputation models, we also analyzed the outcomes in people without missing data (complete-case analysis) and compared the results with those based on the five datasets with imputation.

**Method S7 Trajectory analysis**

We estimated the proportion of smoking, physical inactivity, obesity, glycated hemoglobin (HbA1c) within target, severe glycemic events (diabetic coma or ketoacidosis), blood pressure within target, triglycerides within target, eGFR (estimated glomerular filtration rate) within target, albuminuria (including microalbuminuria and macroalbuminuria), and insulin pump use in people with type 1 diabetes (T1D) at diabetes diagnosis and over diabetes duration according to age at diagnosis (18-29, 30-39, and ≥40 years). Generalized linear models were fitted with adjustment for sex and calendar year at diabetes diagnosis, with logit link function and binomial distribution, and with cluster robust standard errors (SEs) to account for the dependence among measurements in the same individual over time.

**Table S1. Diabetes duration at the time of measurement of prognostic factors**

| **Prognostic factors** | **Diabetes duration (years) at measurement*** |
| --- | --- |
| **Smoking** | 1.47 (1.19, 2.20) |
| **Physical activity** | 1.46 (1.19, 2.11) |
| **BMI** | 1.49 (1.19, 2.26) |
| **HbA1c** | 1.48 (1.19, 2.26) |
| **Systolic blood pressure** | 1.49 (1.20, 2.29) |
| **Diastolic blood pressure** | 1.49 (1.20, 2.28) |
| **Triglycerides** | 1.53 (1.21, 2.36) |
| **eGFR** | 1.51 (1.20, 2.34) |
| **Albuminuria** | 1.51 (1.20, 2.28) |

BMI: body mass index; eGFR: estimated glomerular filtration rate.

*Median (interquartile range) values are presented. The first measurement since cohort entry was used. If that measurement was within 1 year of diabetes diagnosis, the first measurement after 1 year of diagnosis was used instead.

**Table S2. Characteristics of people with type 1 diabetes by sex**

| **Characteristics** | **Men with T1D according to age at diagnosis** | | |  | **Women with T1D according to age at diagnosis** | | |
| --- | --- | --- | --- | --- | --- | --- | --- |
|  | **18-29 years** | **30-39 years** | **≥40 years** |  | **18-29 years** | **30-39 years** | **≥40 years** |
| N | 2554 | 1352 | 2101 |  | 1559 | 820 | 1798 |
| Country of birth in Sweden,% | 95.3 | 91.3 | 91.2 |  | 96 | 93 | 91.4 |
| Married, % | 4.2 | 31.7 | 51.5 |  | 6.7 | 36.5 | 49.6 |
| Post-secondary or higher education,% | 36.2 | 36.7 | 30 |  | 47.9 | 51.5 | 35.2 |
| Smoking*,% | 12.2 | 16.8 | 18.5 |  | 13.8 | 13.4 | 16.9 |
| Physically inactive*,% | 4.5 | 6.8 | 8.6 |  | 4.8 | 5.9 | 3.3 |
| BMI*, mean (SD) | 23.3 (3.9) | 24.7 (4.0) | 25.4 (3.8) |  | 23.6 (5.0) | 24.7 (5.3) | 25.0 (4.8) |
| HbA1c*, median (IQR) | 67.0 (54.0, 85.0) | 70.0 (55.0, 88.0) | 72.0 (57.0, 89.0) |  | 66.0 (53.0, 83.0) | 63.0 (51.0, 80.0) | 72.0 (56.0, 91.0) |
| Systolic blood pressure*, median (IQR) | 120.0 (110.0, 125.0) | 120.0 (115.0, 130.0) | 128.0 (120.0, 137.0) |  | 115.0 (110.0, 120.0) | 115.0 (110.0, 123.0) | 125.0 (115.0, 139.0) |
| Triglycerides*, median (IQR) | 1.0 (0.7, 1.4) | 1.2 (0.8, 1.8) | 1.3 (0.9, 2.0) |  | 0.9 (0.7, 1.2) | 0.9 (0.6, 1.2) | 1.1 (0.9, 1.7) |
| eGFR*, median (IQR) | 119.8 (104.9, 138.9) | 109.6 (95.8, 128.0) | 98.4 (86.0, 114.2) |  | 117.2 (102.1, 138.8) | 106.8 (93.4, 126.2) | 93.5 (79.3, 110.4) |
| Albuminuria*, % | 2.2 | 3.5 | 7.5 |  | 1.6 | 2.3 | 8.8 |
| Diabetic coma or ketoacidosis*, % | 14.5 | 8.2 | 6.9 |  | 15.0 | 7.8 | 10.1 |
| Insulin pump use**, % | 21.3 | 17.6 | 8.0 |  | 26.6 | 34.2 | 20.1 |

T1D: type 1 diabetes; BMI: body mass index; SD: standard deviation; HbA1c: glycated hemoglobin; IQR: interquartile range; eGFR: estimated glomerular filtration rate.

* Measured within 3 months of diabetes diagnosis.

** At 12 years of diabetes duration.

**Table S3. Baseline characteristics of people with type 2 diabetes according to age at diagnosis**

| **Baseline characteristics** | **T2D according to age at diagnosis** | | |
| --- | --- | --- | --- |
|  | **18-29 years** | **30-39 years** | **≥40 years** |
| N | 2,985 | 11,280 | 361,258 |
| Men, s% | 47.9 | 57.1 | 57.7 |
| Born in Sweden,% | 79.6 | 70.1 | 83.2 |
| Married, % | 15.2 | 34.4 | 51.9 |
| Post-secondary or higher education,% | 23.5 | 25.1 | 20.5 |
| Smoking*,% | 27.4 | 24.1 | 15.9 |
| Physically inactive*,% | 17.8 | 17.7 | 14.7 |
| BMI*, mean (SD) | 37.7 (7.8) | 35.6 (7.4) | 30.9 (5.7) |
| HbA1c*, median (IQR) | 56.0 (46.0, 74.0) | 55.0 (46.0, 74.0) | 49.0 (44.0, 60.0) |
| Systolic blood pressure*, median (IQR | 126.0 (120.0, 137.0) | 130.0 (120.0, 139.0) | 135.0 (126.0, 145.0) |
| Triglycerides*, median (IQR) | 2.1 (1.4, 3.2) | 2.1 (1.4, 3.1) | 1.7 (1.2, 2.4) |
| eGFR | 120.0 (105.1, 139.3) | 108.7 (94.4, 125.7) | 83.0 (69.6, 97.6) |
| Albuminuria, % | 16.4 | 16.8 | 16.1 |

T2D: type 2 diabetes; BMI: body mass index; SD: standard deviation; HbA1c: glycated hemoglobin; IQR: interquartile range; eGFR: estimated glomerular filtration rate.

* Measured within 3 months of diabetes diagnosis.

**Table S4. Number of participants and events for different outcomes according to diabetes status and age at diabetes diagnosis/matching**

| **Diabetes status & age at diagnosis /matching** | **Mortality** | | | | | | | | | | | | | | | | | |  | **MACE** | | |
| --- | --- | --- | --- | --- | --- | --- | --- | --- | --- | --- | --- | --- | --- | --- | --- | --- | --- | --- | --- | --- | --- | --- |
|  | **Participants** | **All-cause**  **mortality** | |  | **Cardiovascular**  **death** | |  | **Non-cardiovascular death** | |  | **Cancer death** | |  | **Death due to infection** | |  | **Death from diabetes coma or ketoacidosis** | |  |  |  |  |
|  |  | No. of events | Incidence rate* |  | No. of events | Incidence rate* |  | No. of events | Incidence rate* |  | No. of events | Incidence rate* |  | No. of events | Incidence rate* |  | No. of events | Incidence rate* |  | Participants | No. of events | Incidence rate* |
| **Controls** |  |  |  |  |  |  |  |  |  |  |  |  |  |  |  |  |  |  |  |  |  |  |
| 18-29 years | 205,649 | 1,291 | 0.6 |  | 55 | 0.03 |  | 1,098 | 0.6 |  | 146 | 0.1 |  | 12 | 0.006 |  | - | - |  | 205,220 | 418 | 0.2 |
| 30-39 years | 108,600 | 1,065 | 0.9 |  | 144 | 0.1 |  | 816 | 0.8 |  | 262 | 0.2 |  | 19 | 0.02 |  | - | - |  | 108,268 | 868 | 0.8 |
| ≥40 years | 194,923 | 24,532 | 12.8 |  | 7,869 | 4.5 |  | 14,781 | 8.4 |  | 6,262 | 3.6 |  | 726 | 0.4 |  | - | - |  | 179,984 | 12,748 | 7.6 |
| **T1D** |  |  |  |  |  |  |  |  |  |  |  |  |  |  |  |  |  |  |  |  |  |  |
| 18-29 years | 4,113 | 50 | 1.2 |  | 1 | 0.0 |  | 41 | 1.0 |  | 4 | 0.1 |  | 1 | 0.03 |  | 18 | 0.5 |  | 4,108 | 15 | 0.4 |
| 30-39 years | 2,172 | 60 | 2.6 |  | 6 | 0.3 |  | 48 | 2.3 |  | 10 | 0.5 |  | 0 | 0.0 |  | 19 | 0.9 |  | 2,170 | 26 | 1.2 |
| ≥40 years | 3,899 | 706 | 19.4 |  | 195 | 5.8 |  | 466 | 13.9 |  | 170 | 5.1 |  | 30 | 0.9 |  | 77 | 2.3 |  | 3,714 | 320 | 9.7 |
| **T2D** |  |  |  |  |  |  |  |  |  |  |  |  |  |  |  |  |  |  |  |  |  |  |
| 18-29 years | 2,985 | 50 | 1.8 |  | 4 | 0.2 |  | 43 | 1.7 |  | 5 | 0.2 |  | 1 | 0.04 |  | 3 | 0.1 |  | 2,971 | 30 | 1.2 |
| 30-39 years | 11,280 | 377 | 3.4 |  | 87 | 0.9 |  | 256 | 2.5 |  | 65 | 0.6 |  | 17 | 0.2 |  | 20 | 0.2 |  | 11,151 | 370 | 3.6 |
| ≥40 years | 361,258 | 90,494 | 30.7 |  | 28,850 | 10.7 |  | 52,335 | 19.4 |  | 20,217 | 7.5 |  | 4,051 | 1.5 |  | 5,285 | 2.0 |  | 314,315 | 47,974 | 20.2 |

MACE: major adverse cardiovascular events; T1D: type 1 diabetes; T2D: type 2 diabetes.

*Number of events per 1000 person years.

**Table S5. Complete-case analysis for the association between prognostic factors and all-cause mortality in adult-onset type 1 diabetes**

| **Lifestyle and clinical characteristics** | **Overall** | | | | |  | **T1D diagnosed at age ≥40 years** | | | | |
| --- | --- | --- | --- | --- | --- | --- | --- | --- | --- | --- | --- |
|  | **Main model** | |  | **Mutually adjusted**  **model** | |  | **Main model** | |  | **Mutually adjusted**  **model** | |
|  | **Sample size*** | **HR (95% CI)** |  | **Sample size*** | **HR (95% CI)** |  | **Sample size**** | **HR (95% CI)** |  | **Sample size**** | **HR (95% CI)** |
| Smoking | 8351 | 2.15 (1.77, 2.63) |  | 4070 | 2.36 (1.70, 3.28) |  | 3057 | 2.10 (1.69, 2.62) |  | 1536 | 2.36 (1.63, 3.41) |
| Being physically inactive vs active | 7208 | 2.01 (1.50, 2.69) |  | 4070 | 1.37 (0.82, 2.31) |  | 2660 | 2.40 (1.77, 3.27) |  | 1536 | 1.92 (1.11, 3.31) |
| BMI | 8040 |  |  | 4070 |  |  | 3003 |  |  | 1536 |  |
| Underweight |  | 2.05 (1.23, 3.41) |  |  | 0.69 (0.10, 5.02) |  |  | 2.03 (1.13, 3.66) |  |  | 1.30 (0.18, 9.64) |
| Overweight |  | 1.00 (0.82, 1.23) |  |  | 0.90 (0.64, 1.27) |  |  | 1.13 (0.91, 1.41) |  |  | 1.02 (0.70, 1.50) |
| Obesity |  | 1.58 (1.25, 2.01) |  |  | 1.52 (1.00, 2.33) |  |  | 1.71 (1.32, 2.21) |  |  | 1.73 (1.09, 2.77) |
| HbA1c out of target | 9166 | 1.38 (1.17, 1.62) |  | 4070 | 1.46 (1.07, 1.99) |  | 3460 | 1.25 (1.05, 1.48) |  | 1536 | 1.33 (0.95, 1.87) |
| Blood pressure out of target | 8601 | 1.24 (1.05, 1.47) |  | 4070 | 1.14 (0.84, 1.56) |  | 3281 | 1.16 (0.96, 1.39) |  | 1536 | 1.12 (0.79, 1.60) |
| Triglycerides out of target | 6379 | 1.67 (1.33, 2.10) |  | 4070 | 1.32 (0.90, 1.93) |  | 2481 | 1.62 (1.27, 2.07) |  | 1536 | 1.07 (0.70, 1.64) |
| eGFR out of target | 7625 | 1.36 (1.07, 1.73) |  | 4070 | 1.36 (0.79, 2.35) |  | 3025 | 1.32 (1.04, 1.68) |  | 1536 | 1.40 (0.81, 2.43) |
| Albuminuria | 7345 | 1.95 (1.54, 2.48) |  | 4070 | 1.75 (1.18, 2.59) |  | 2728 | 1.99 (1.55, 2.54) |  | 1536 | 1.73 (1.14, 2.62) |

HR: hazard ratio; CI: confidence interval; BMI: body mass index; HbA1c: glycated hemoglobin; eGFR: estimated glomerular filtration rate

The basic models were fitted with attained age as the time scale, with adjustment for age and year at diabetes diagnosis, diabetes duration at NDR visit, country of birth, education, marital status, and with stratification by sex. The full models were additionally adjusted for all variables in the table, while other models remove some covariates based on the full models.

*Total sample size in the imputed dataset: 9493.

**Total sample size in the imputed dataset: 3588.

**Table S6. Number of participants and events for different outcomes in population controls and people with T1D according to number of risk factors**

| **Exposure** | **Mortality** | | | | | |  | **MACE** | |
| --- | --- | --- | --- | --- | --- | --- | --- | --- | --- |
|  | **Participants** | **All-cause mortality** | **Cardiovascular death** | **Non-cardiovascular death** | **Cancer death** | **Death due to infection** |  | **Participants** | **Events** |
| **Population controls** | 469539 | 21978 | 6304 | 13681 | 5482 | 649 |  | 454406 | 11726 |
| **People with T1D (dataset with imputation 1)*** | | | | | | | | | |
| No risk factor | 2205 | 34 | 5 | 23 | 12 | 0 |  | 2191 | 24 |
| 1 risk factor | 3264 | 139 | 23 | 105 | 43 | 10 |  | 3232 | 57 |
| 2 risk factors | 2254 | 190 | 46 | 128 | 42 | 9 |  | 2194 | 78 |
| At least 3 risk factors | 1770 | 396 | 117 | 257 | 68 | 11 |  | 1678 | 161 |
| **People with T1D (dataset with imputation 2)*** | | | | | | | | | |
| No risk factor | 2217 | 34 | 6 | 22 | 12 | 1 |  | 2204 | 26 |
| 1 risk factor | 3266 | 141 | 24 | 103 | 45 | 6 |  | 3231 | 59 |
| 2 risk factors | 2263 | 205 | 49 | 136 | 38 | 9 |  | 2200 | 84 |
| At least 3 risk factors | 1747 | 379 | 112 | 252 | 70 | 14 |  | 1660 | 151 |
| **People with T1D (dataset with imputation 3)*** | | | | | | | | | |
| No risk factor | 2210 | 35 | 4 | 26 | 13 | 1 |  | 2197 | 21 |
| 1 risk factor | 3315 | 138 | 26 | 102 | 47 | 6 |  | 3278 | 62 |
| 2 risk factors | 2251 | 208 | 51 | 137 | 37 | 13 |  | 2194 | 94 |
| At least 3 risk factors | 1717 | 378 | 110 | 248 | 68 | 10 |  | 1626 | 143 |
| **People with T1D (dataset with imputation 4)*** | | | | | | | | | |
| No risk factor | 2200 | 34 | 5 | 24 | 12 | 0 |  | 2186 | 22 |
| 1 risk factor | 3297 | 145 | 24 | 110 | 45 | 10 |  | 3264 | 63 |
| 2 risk factors | 2246 | 170 | 42 | 107 | 31 | 8 |  | 2195 | 80 |
| At least 3 risk factors | 1750 | 410 | 120 | 272 | 77 | 12 |  | 1650 | 155 |
| **People with T1D (dataset with imputation 5)*** | | | | | | | | | |
| No risk factor | 2216 | 40 | 6 | 28 | 15 | 1 |  | 2202 | 25 |
| 1 risk factor | 3256 | 135 | 20 | 105 | 42 | 5 |  | 3224 | 62 |
| 2 risk factors | 2284 | 209 | 51 | 140 | 37 | 10 |  | 2225 | 87 |
| At least 3 risk factors | 1737 | 375 | 114 | 240 | 71 | 14 |  | 1644 | 146 |

MACE: major adverse cardiovascular events; T1D: type 2 diabetes; *For people with T1D, the multivariate imputation by chained equations (MICE) algorithm imputed missing data for risk factors and derived 5 datasets.

**Table S7. Distribution of prognostic factors in people with T1D according to age at diagnosis and educational levels**

| **Prognostic factors** | **Post-secondary or higher education** | | |  | **Lower than post-secondary** | | |
| --- | --- | --- | --- | --- | --- | --- | --- |
|  | **18-29 years** | **30-39 years** | **≥40 years** |  | **18-29 years** | **30-39 years** | **≥40 years** |
| Smoking*, % | 5.7 | 7.5 | 8.2 |  | 16.8 | 21.2 | 23.2 |
| Physically inactive*, % | 3.5 | 5.0 | 4.0 |  | 5.1 | 7.4 | 7.1 |
| BMI*, mean (SD) | 22.7 (3.7) | 23.7 (3.9) | 24.7 (4.1) |  | 23.8 (4.6) | 25.4 (4.7) | 25.4 (4.4) |
| HbA1c*, median (IQR) | 65.0 (52.0, 82.0) | 64.0 (50.0, 79.0) | 72.0 (56.0, 90.0) |  | 67.0 (55.0, 85.0) | 71.0 (56.0, 89.0) | 73.0 (57.0, 89.0) |
| Systolic blood pressure*, median (IQR) | 118.0 (110.0, 124.0) | 118.0 (110.0, 125.0) | 122.0 (115.0, 133.0) |  | 118.5 (110.0, 125.0) | 120.0 (113.0, 130.0) | 130.0 (120.0, 140.0) |
| Triglycerides*, median (IQR) | 0.9 (0.7, 1.2) | 0.9 (0.7, 1.3) | 1.1 (0.8, 1.7) |  | 1.0 (0.7, 1.3) | 1.2 (0.9, 1.9) | 1.3 (0.9, 1.9) |
| eGFR*, median (IQR) | 118.9 (102.1, 137.0) | 103.9 (93.3, 123.1) | 95.5 (81.9, 109.1) |  | 119.3 (104.0, 140.3) | 112.8 (97.1, 130.6) | 97.0 (83.3, 114.2) |
| Albuminuria*, % | 1.6 | 1.7 | 6.9 |  | 2.2 | 4.1 | 8.8 |
| Diabetic coma or ketoacidosis*, % | 0.2 | 0.8 | 0.2 |  | 13.8 | 8.0 | 8.8 |
| Insulin pump use**, % | 29.2 | 27.6 | 18.5 |  | 18.1 | 22.1 | 11.0 |

BMI: body mass index; SD: standard deviation; HbA1c: glycated hemoglobin; IQR: interquartile range; eGFR: estimated glomerular filtration rate.

* Measured within 3 months of diabetes diagnosis

** At 12 years of diabetes duration.


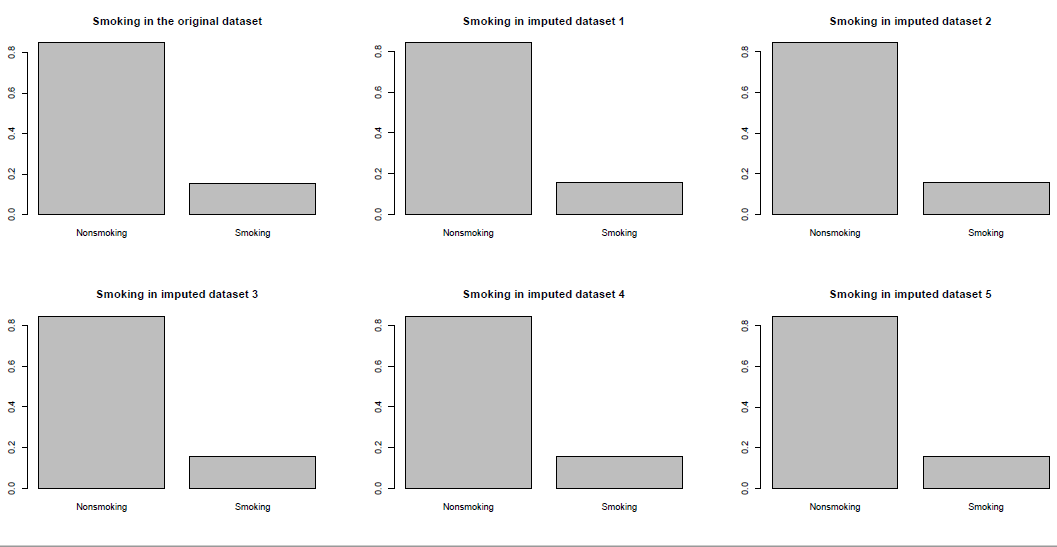


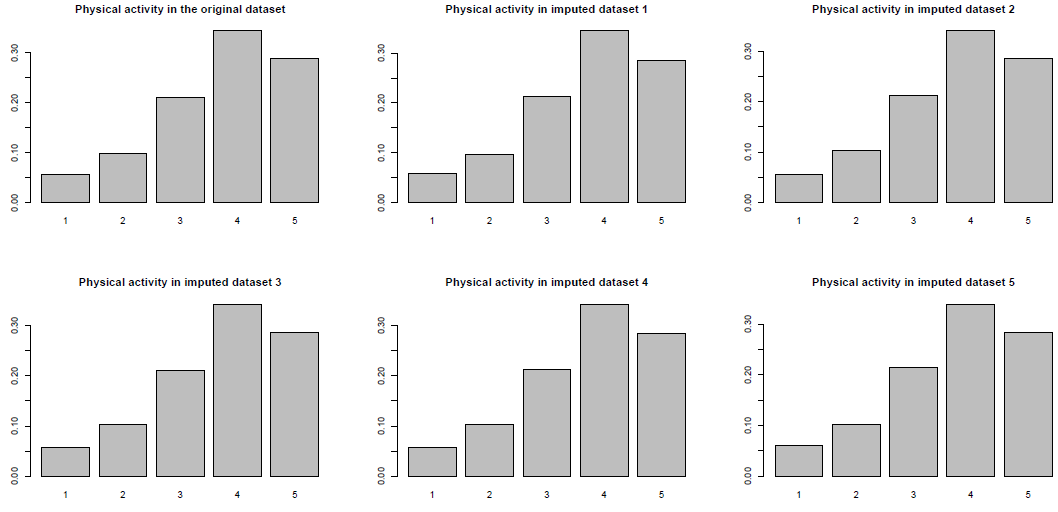


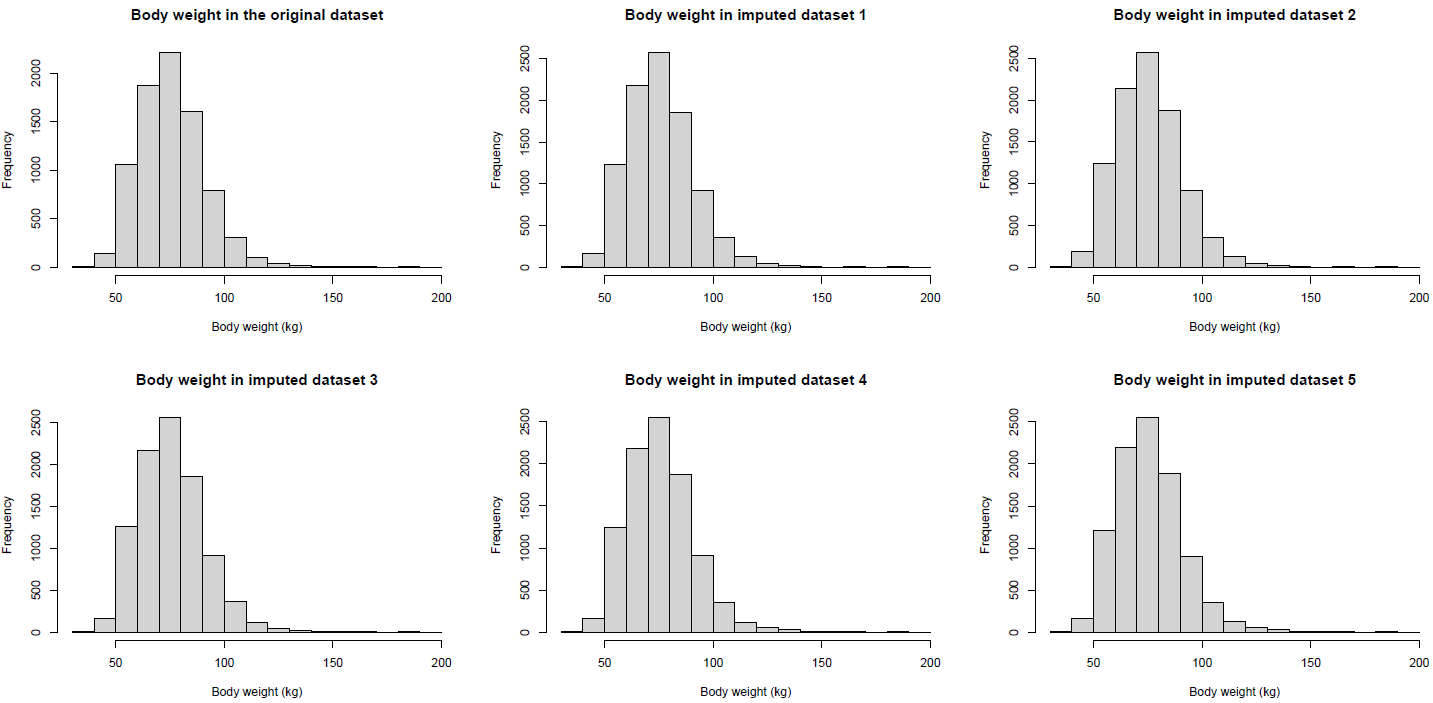


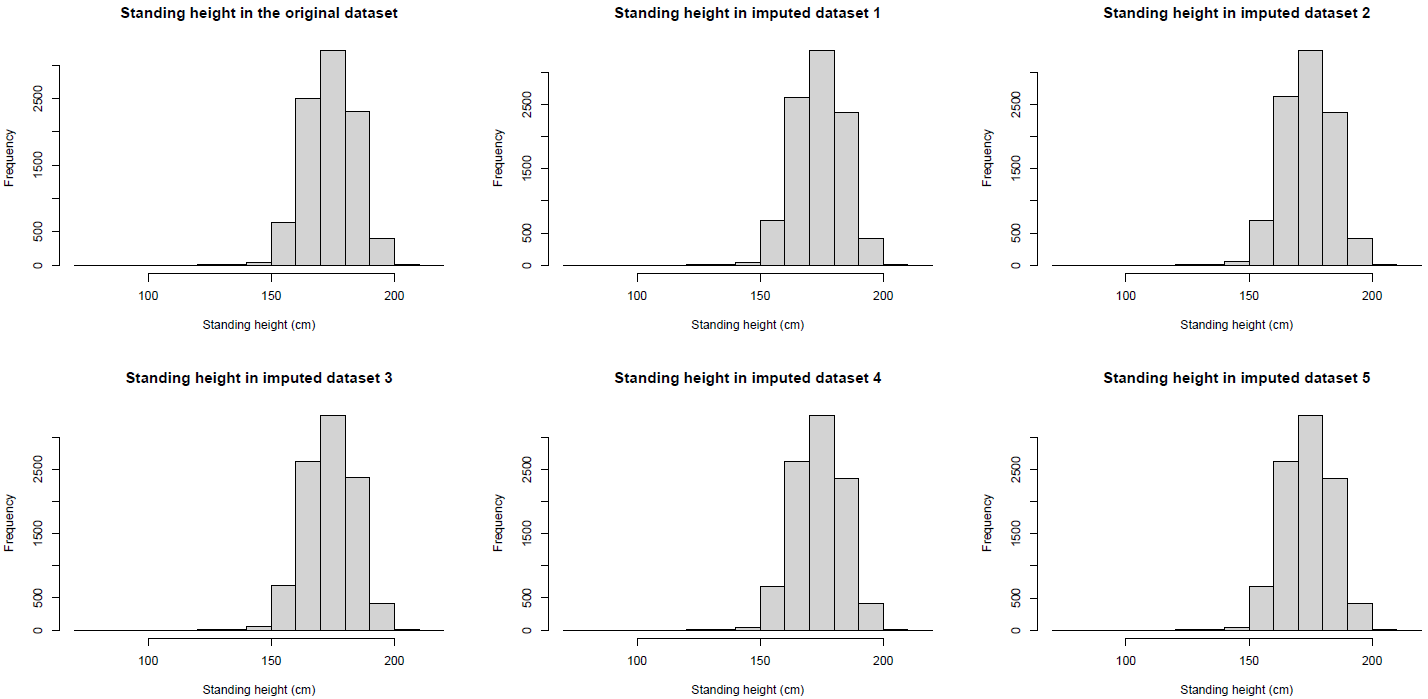


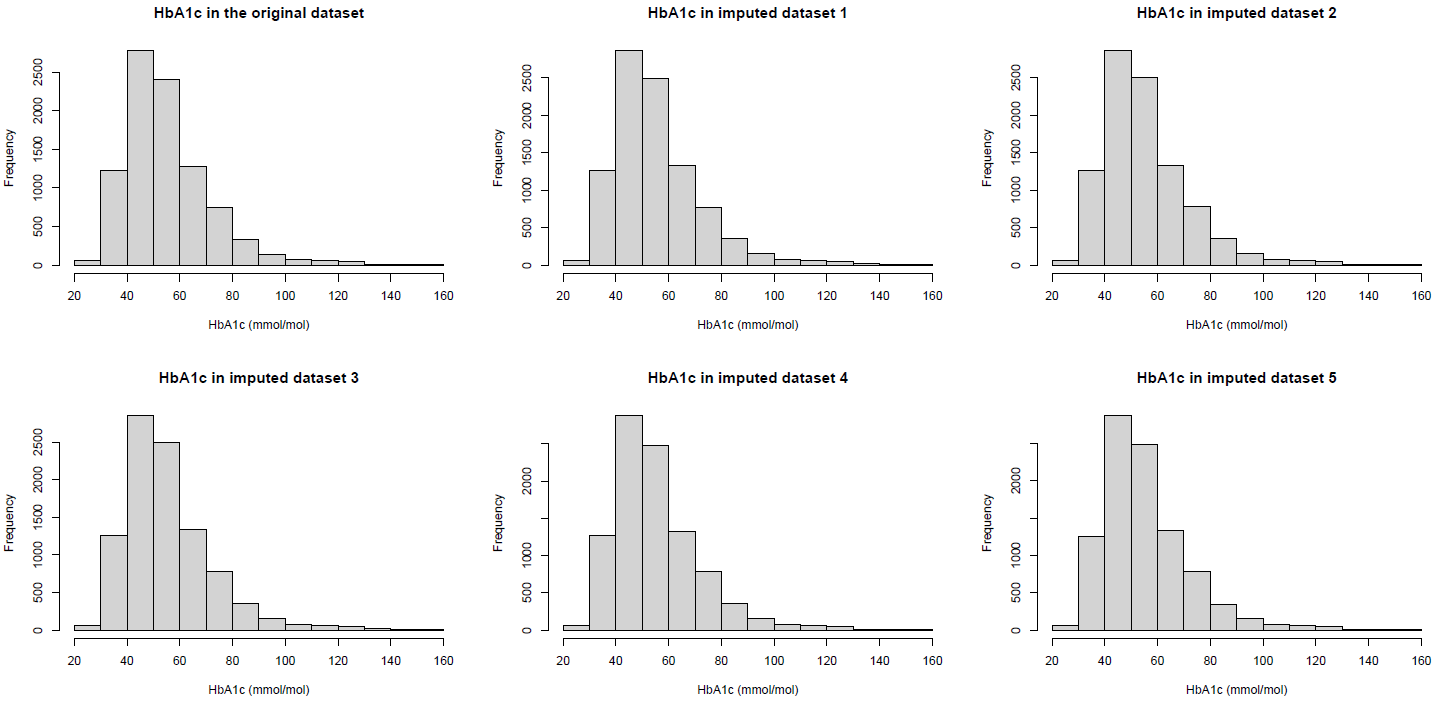


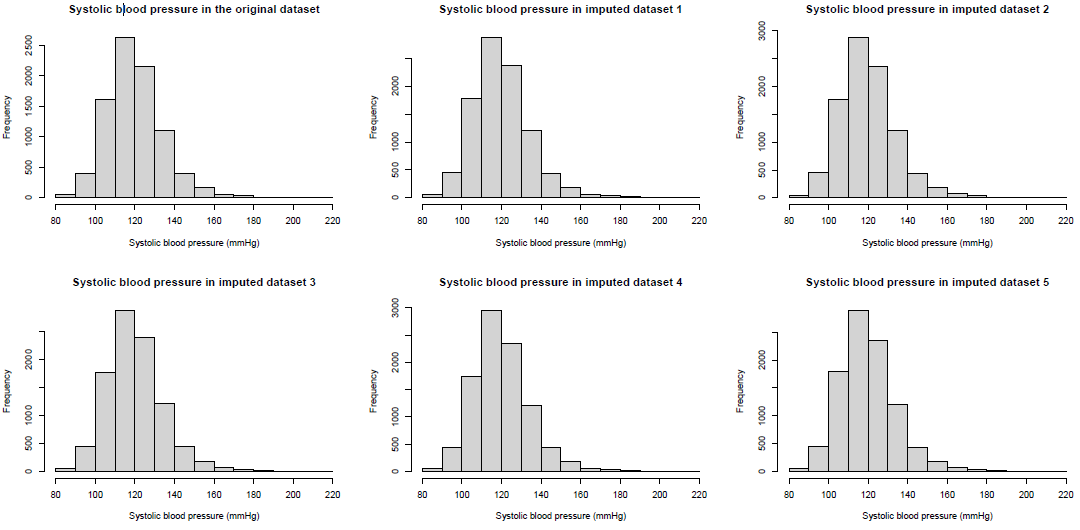


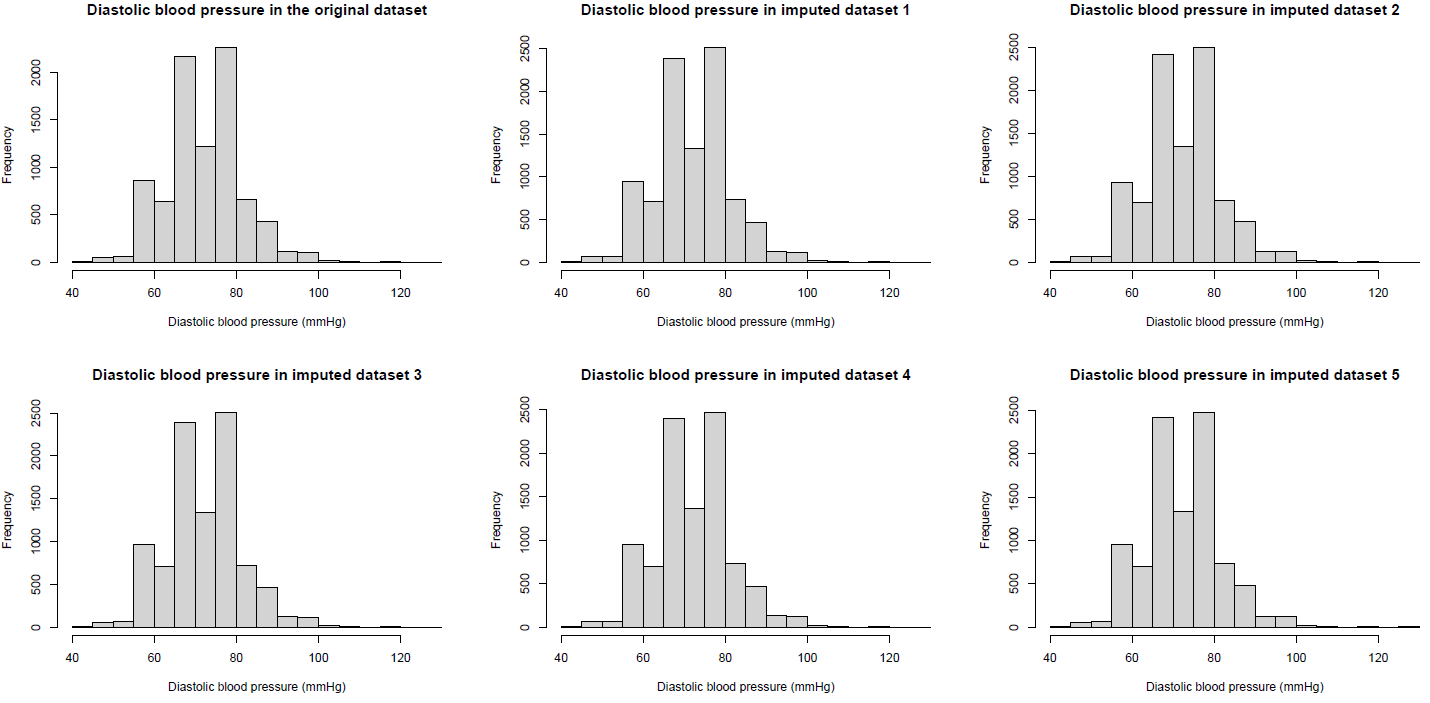


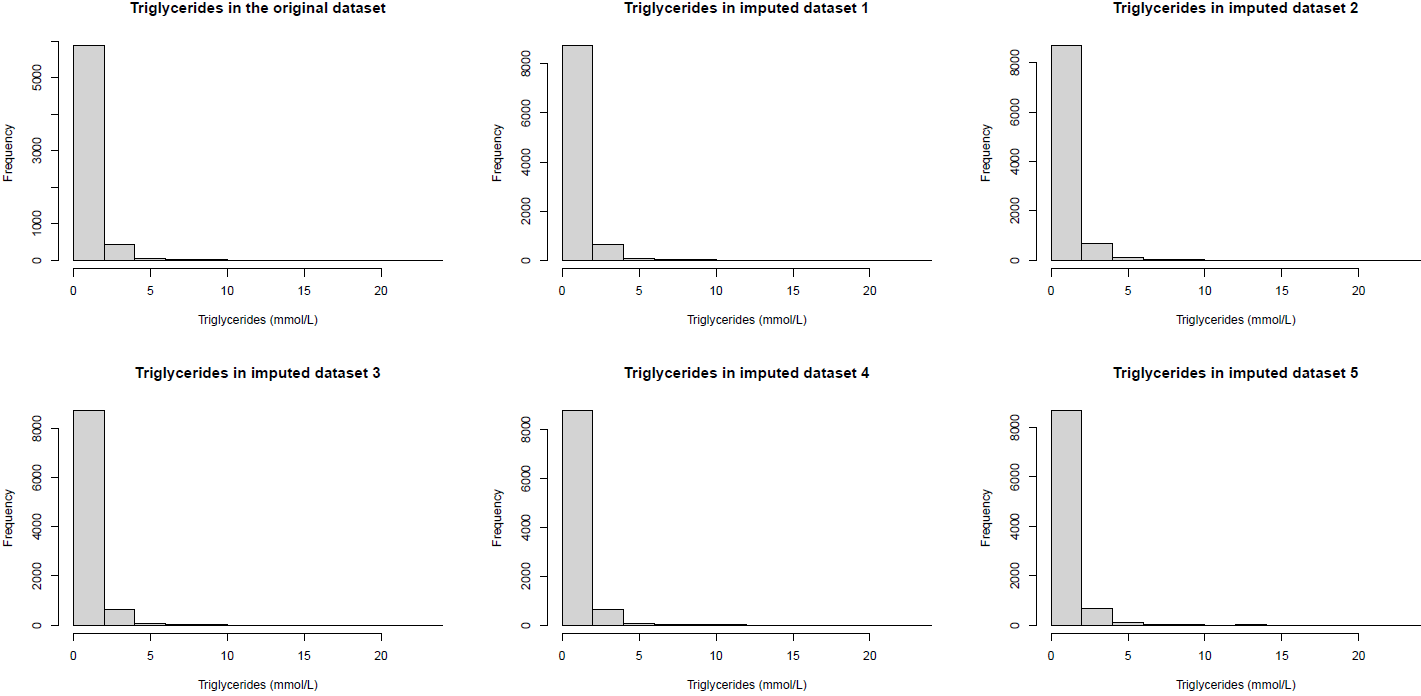


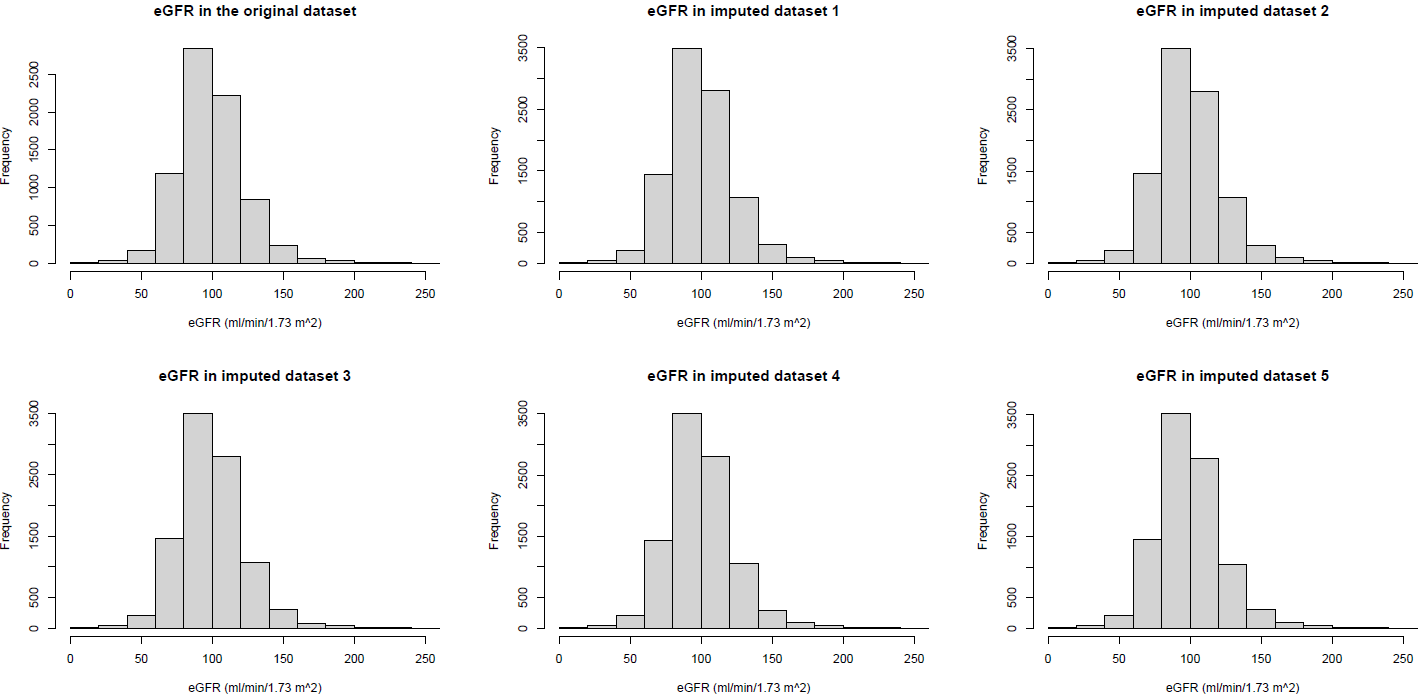


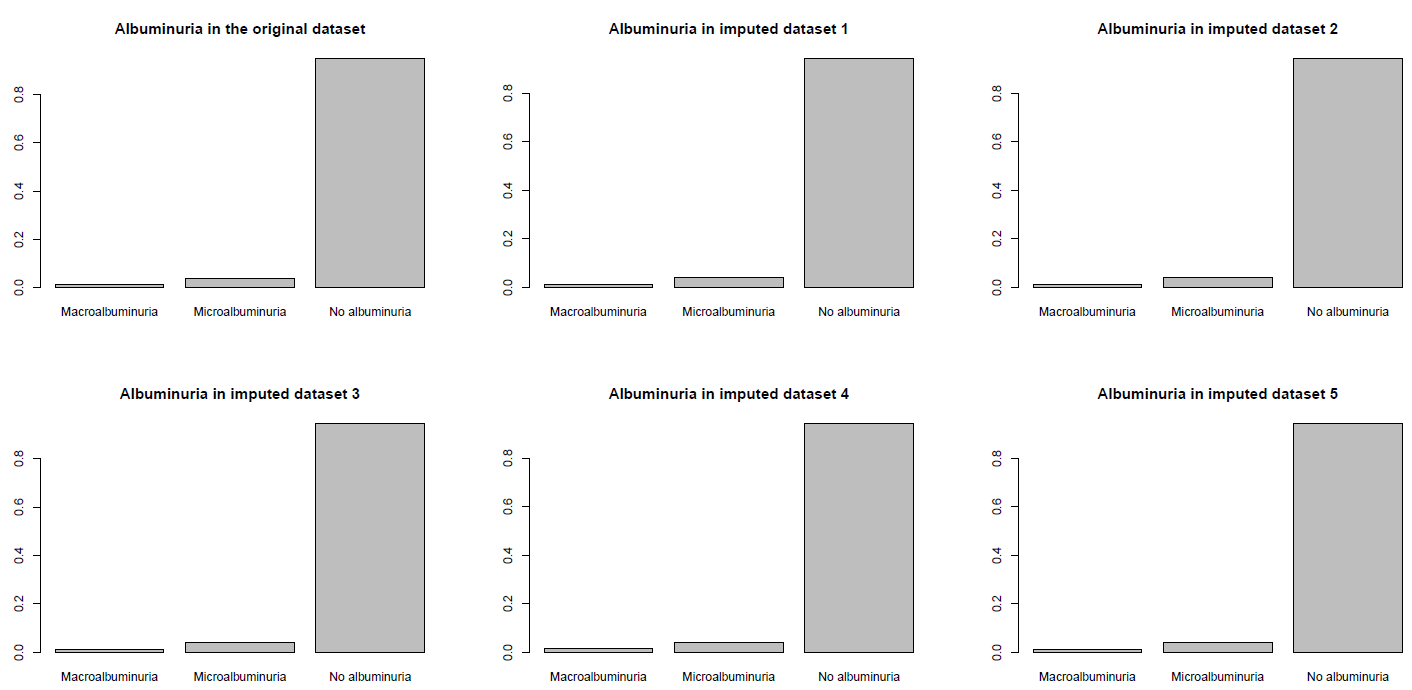


**Figure S1. Distribution of variables in the original dataset and the five datasets with multivariate imputation by chained equations**

HbA1c: glycated hemoglobin; eGFR: estimated glomerular filtration rate

Categories of physical activity (30 minutes of walk or equivalent): 1 represents never, 2 represents <1 time/week, 3 represents 1-2 times/week, 4 represents 3-5 times/week; 5 represents daily.

The missing rate was 12% for smoking, 24% for physical activity, 14% for body weight, 3% for HbA1c, 9% for blood pressure, 33% for triglycerides, 20% for eGFR, and 23% for albuminuria.


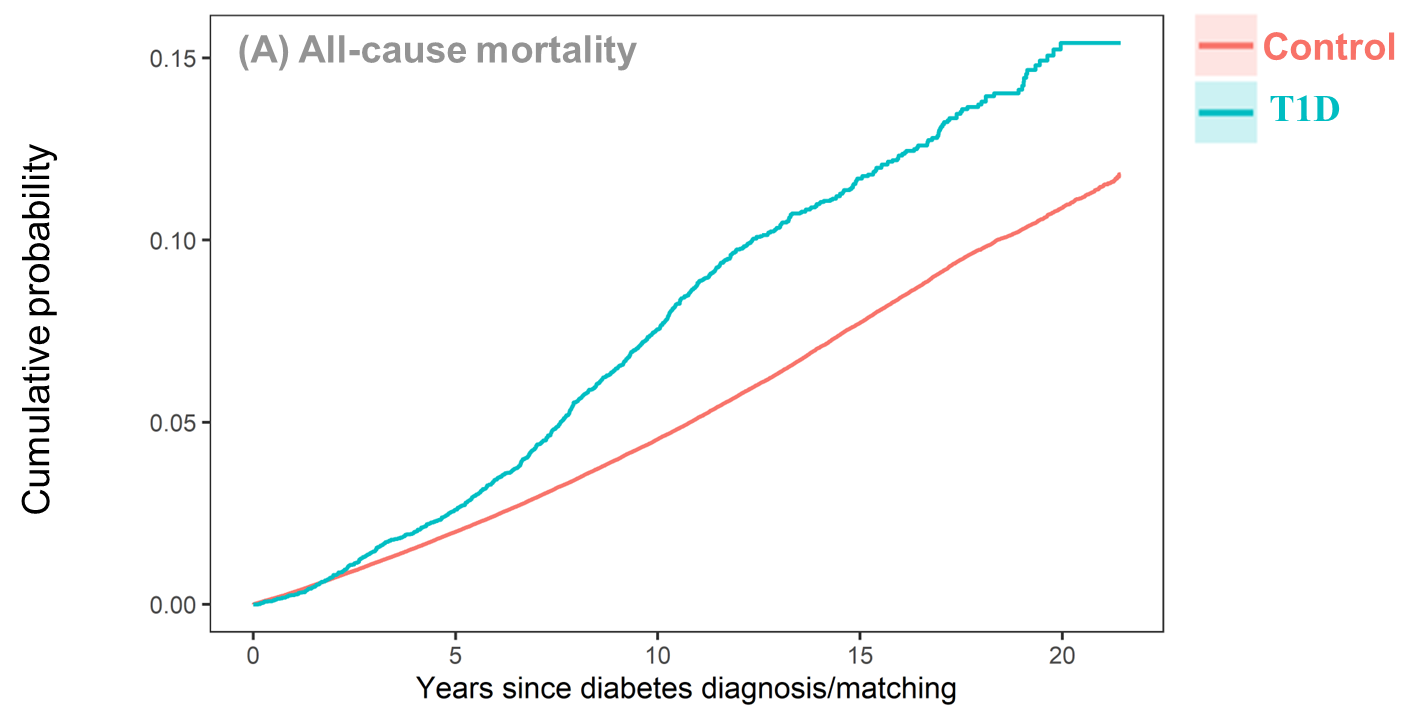


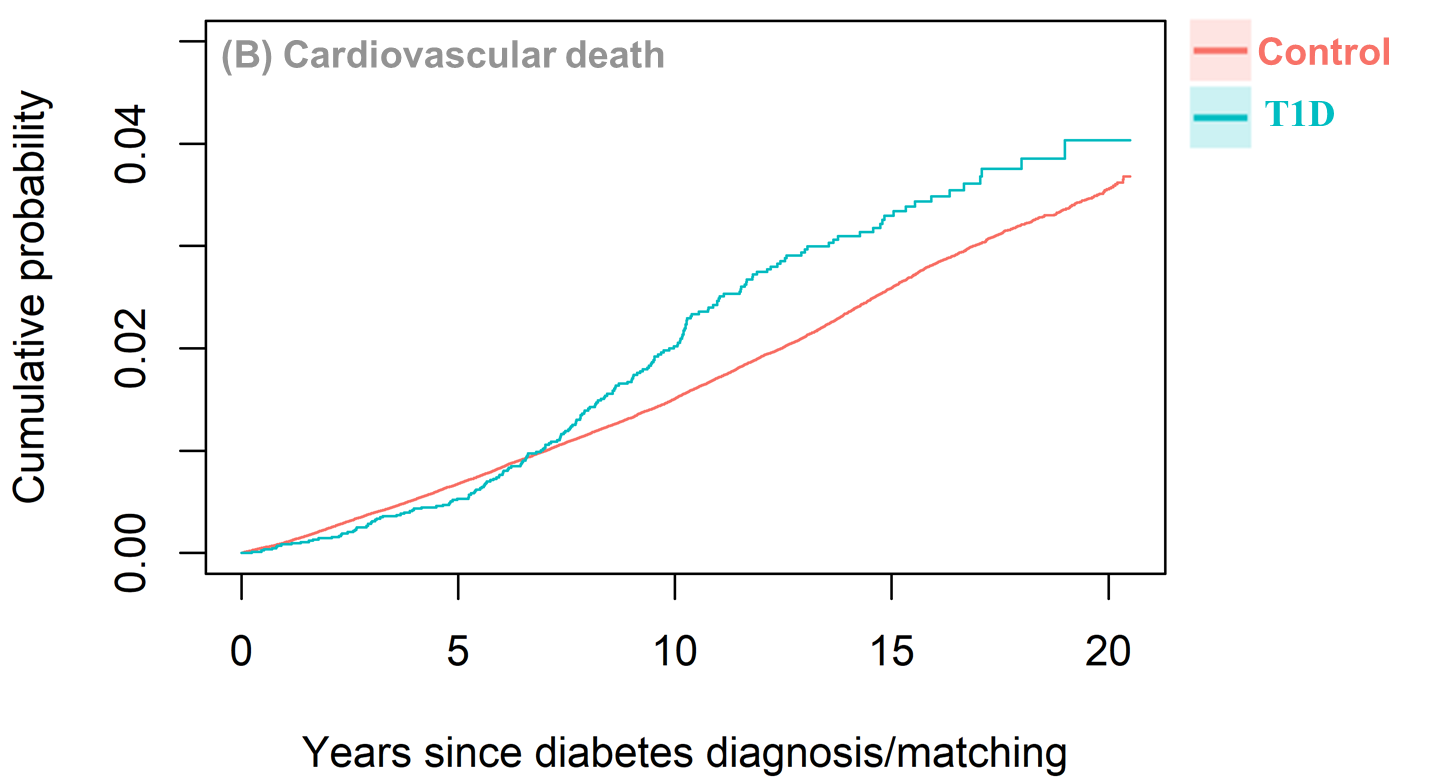


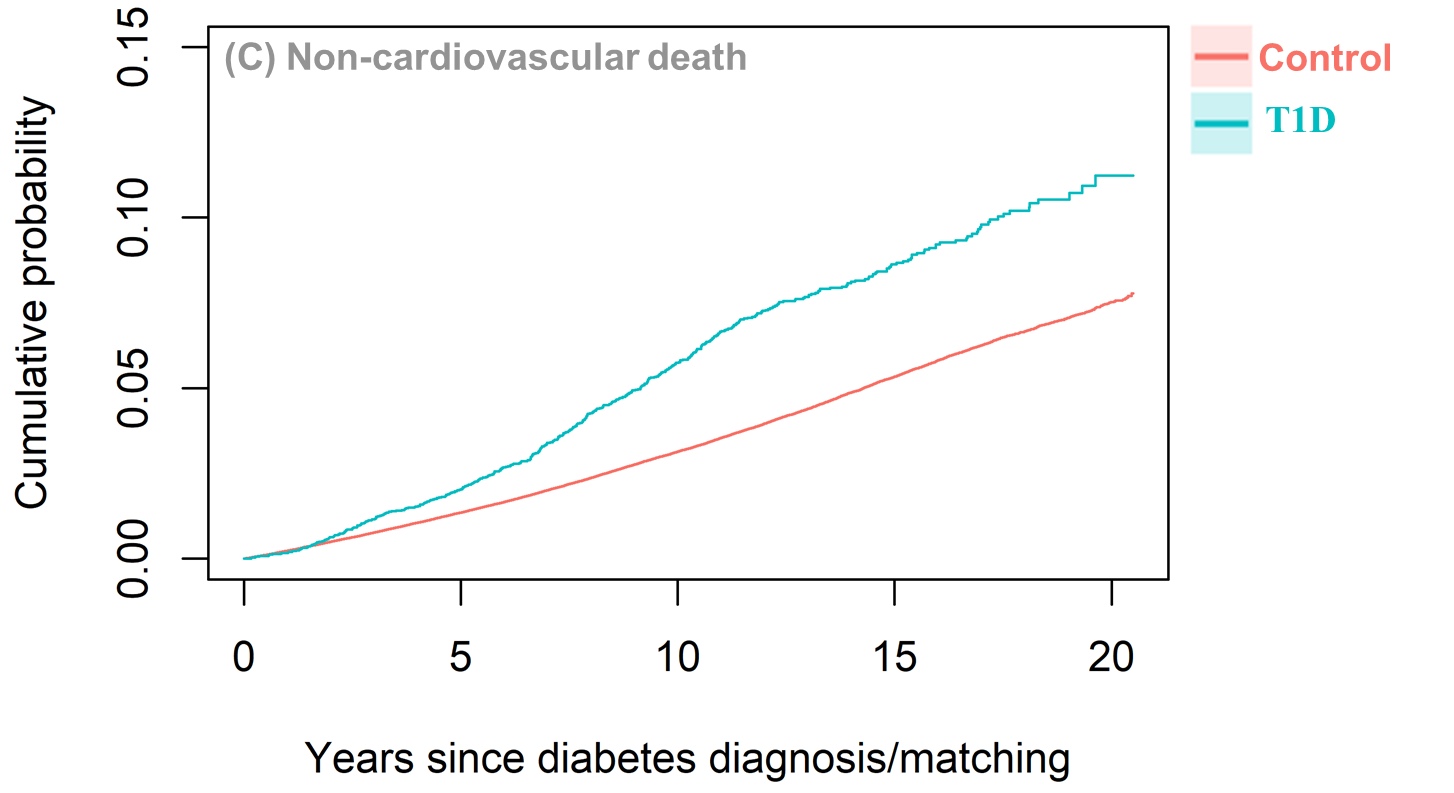


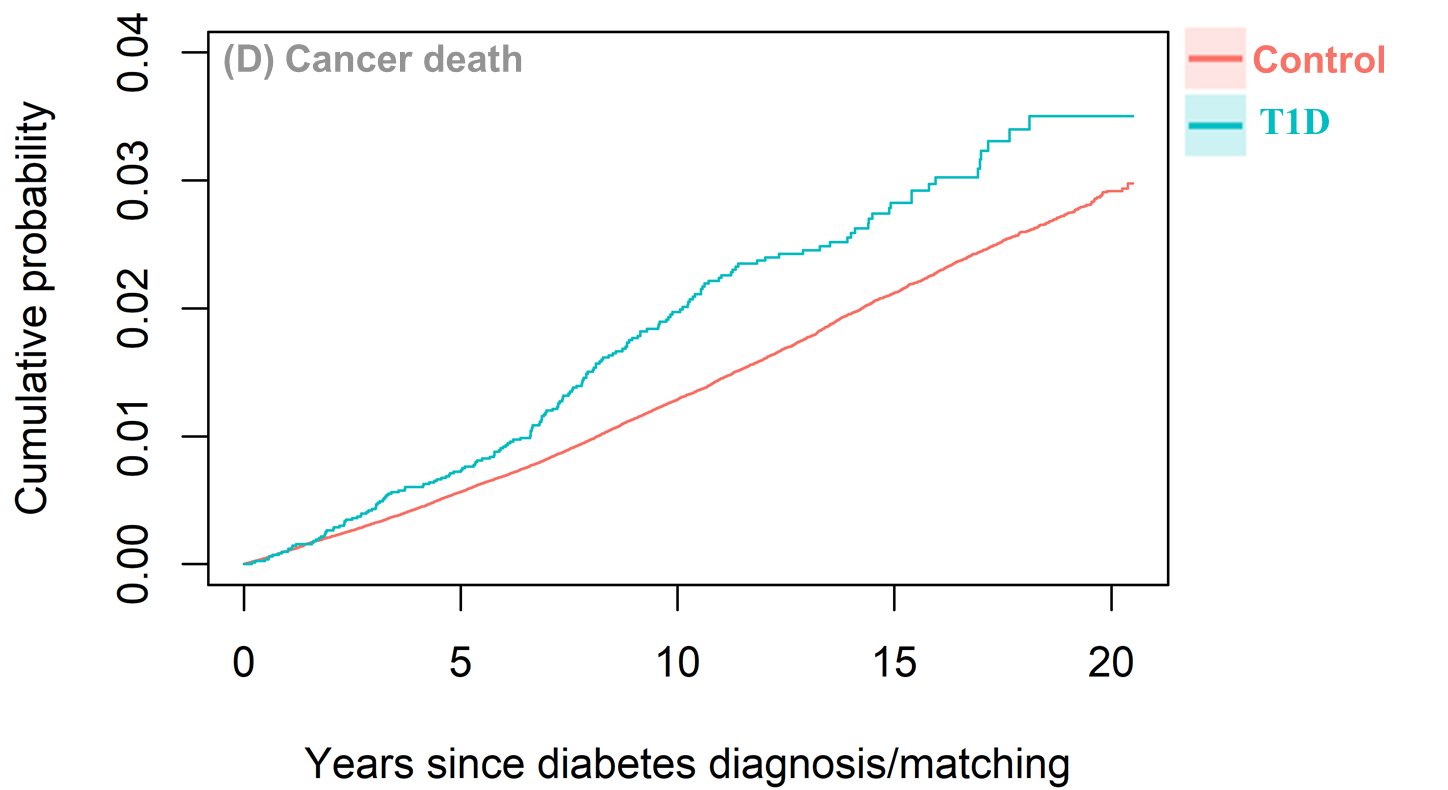


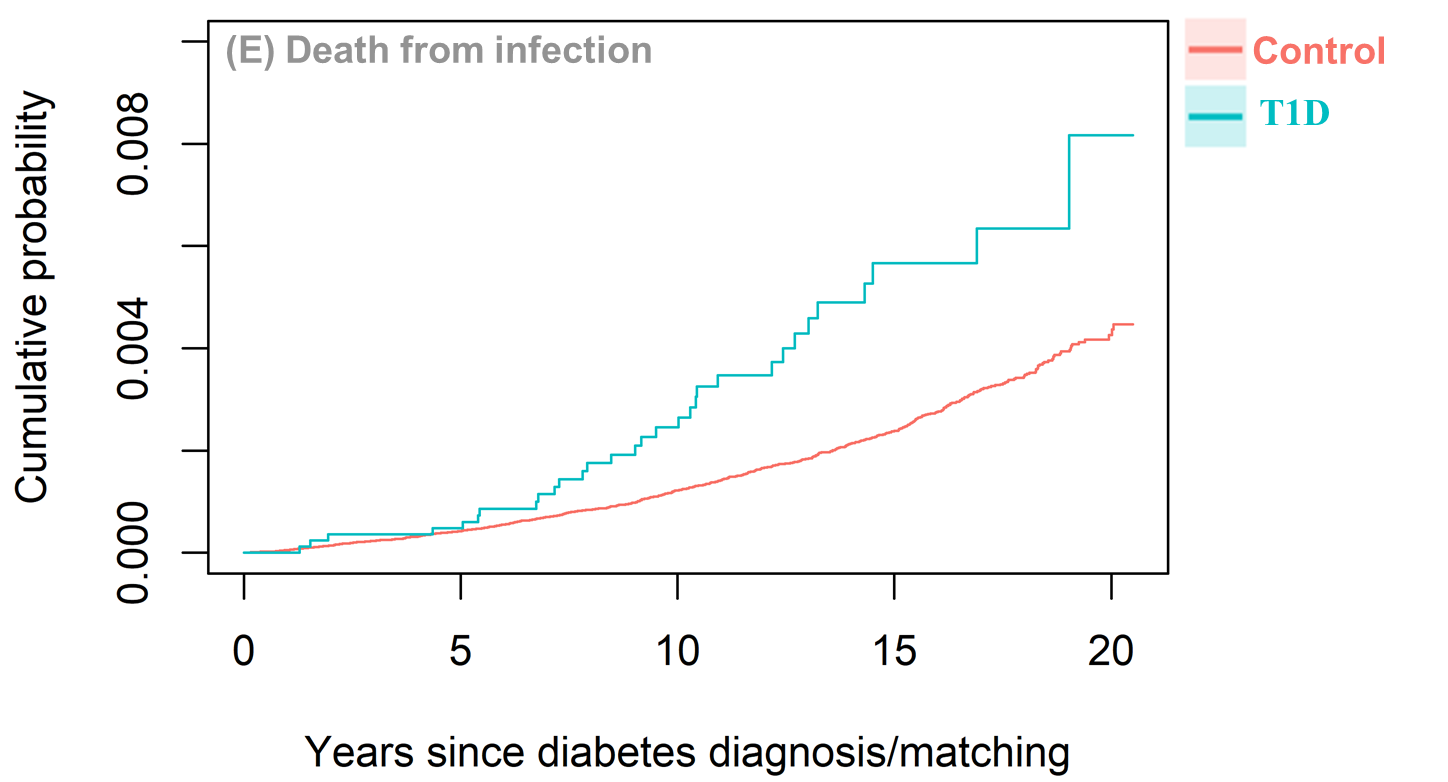


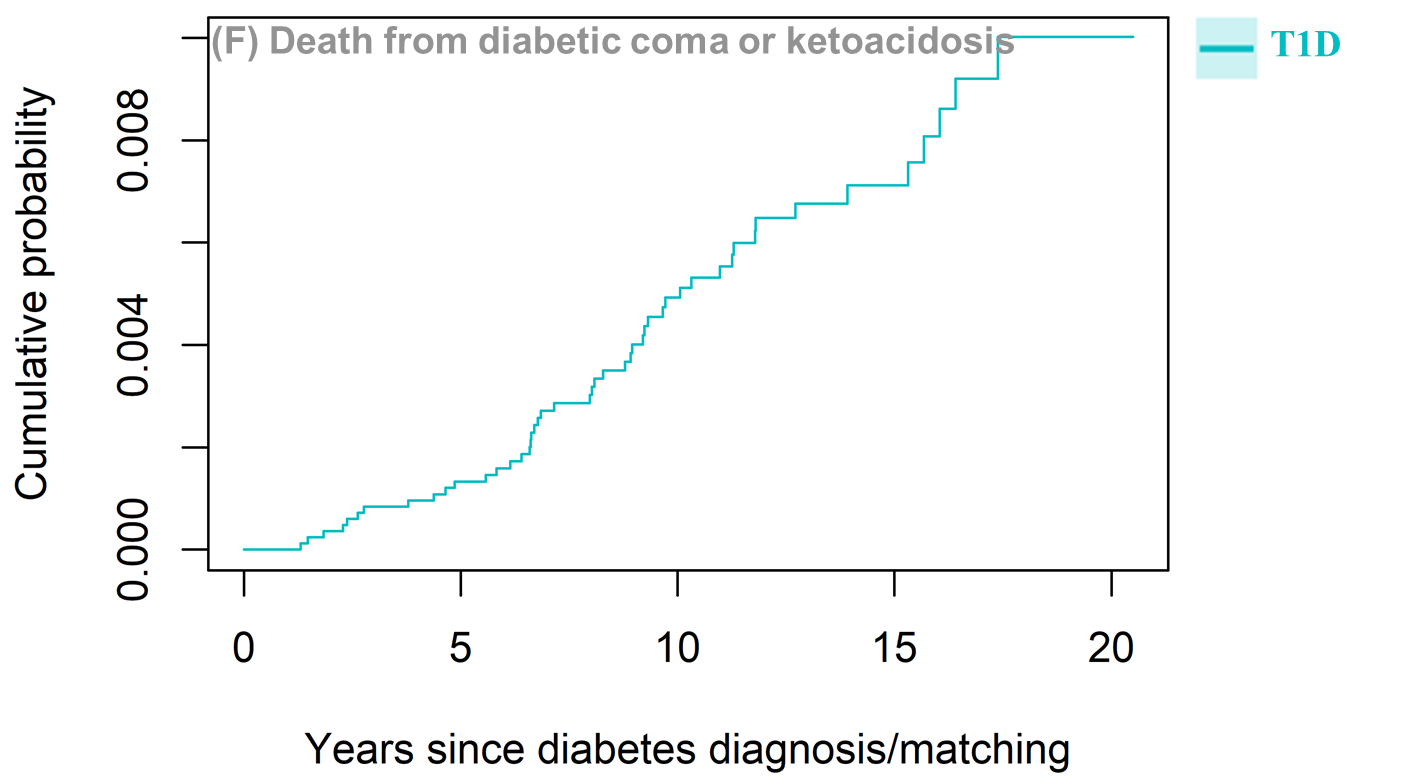


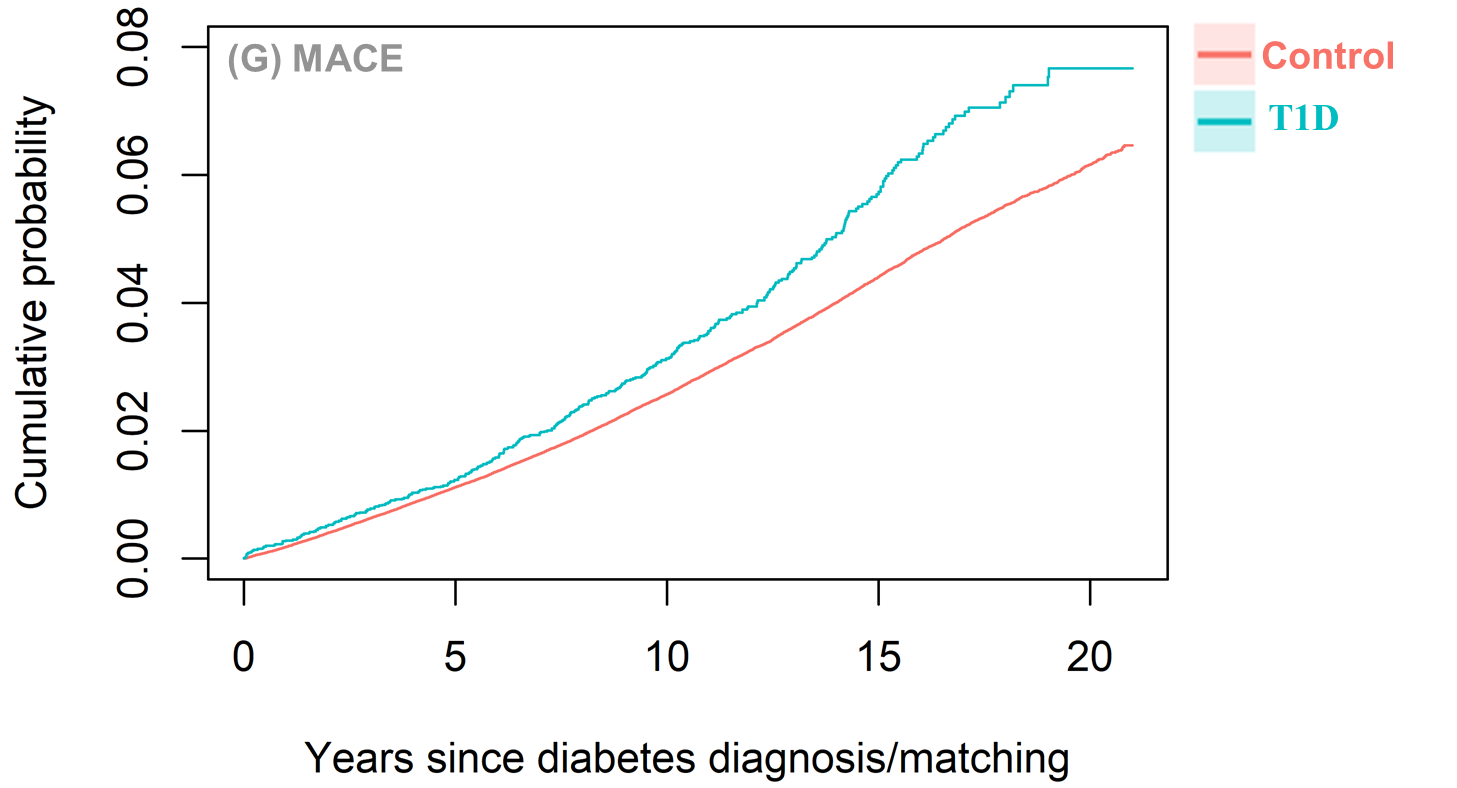


**Figure S2. Cumulative probability of different outcomes in T1D as compared to population controls over diabetes/matching duration**

MACE: major adverse cardiovascular events; T1D: type 1 diabetes.

Cumulative probability of all-cause mortality was plotted using the Kaplan-Meier approach. Cumulative probability of cause-specific mortality and MACE was plotted based on the cumulative incidence function using the Aalen-Johansen method to account for competing events.


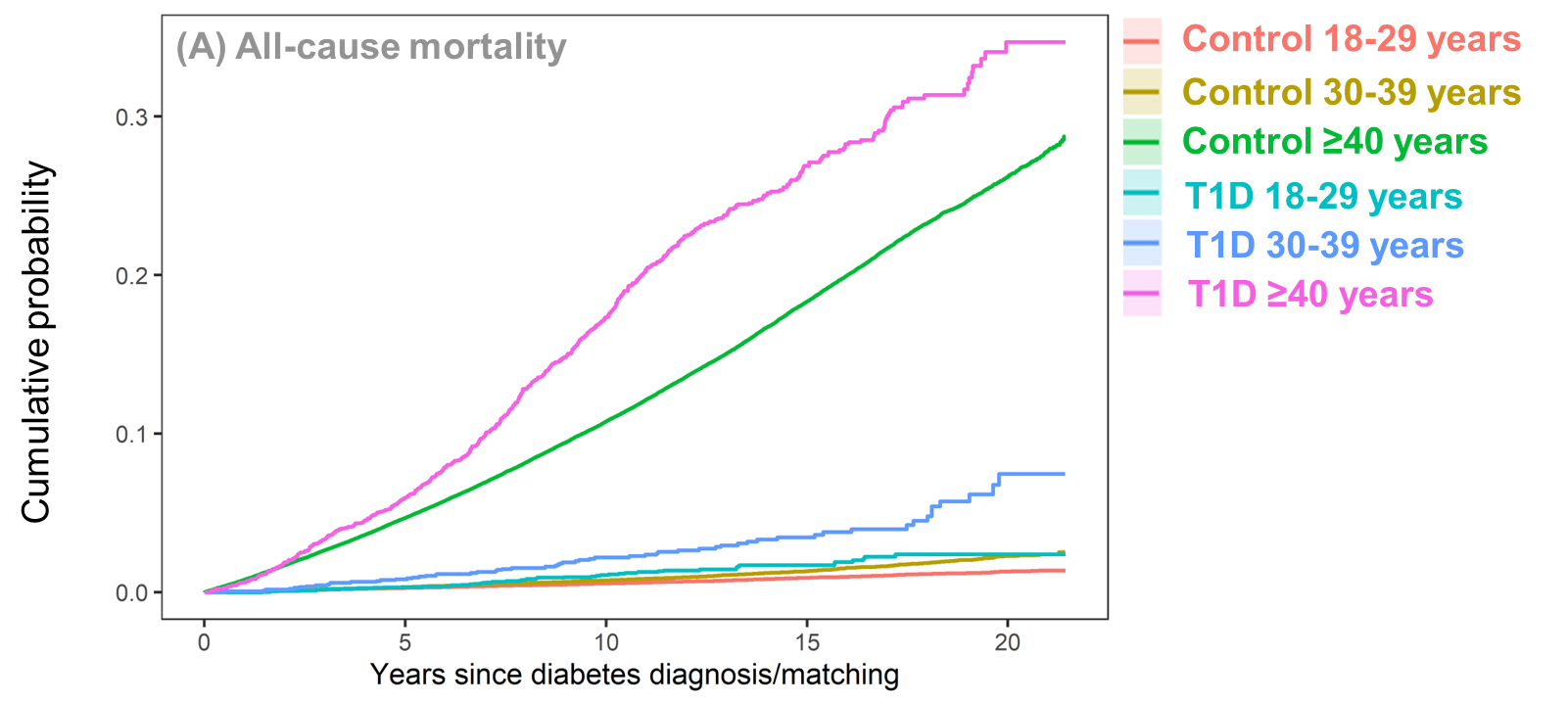


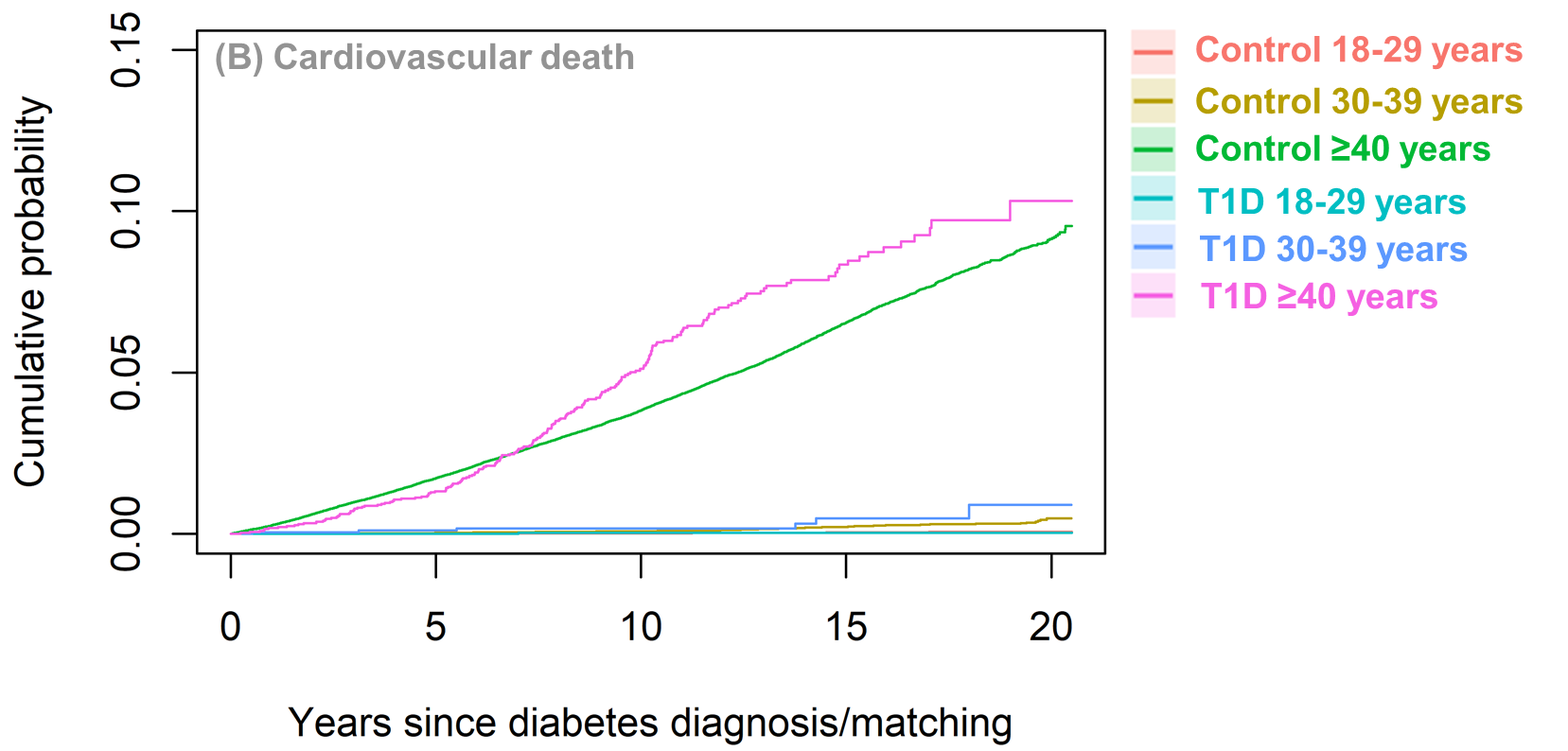


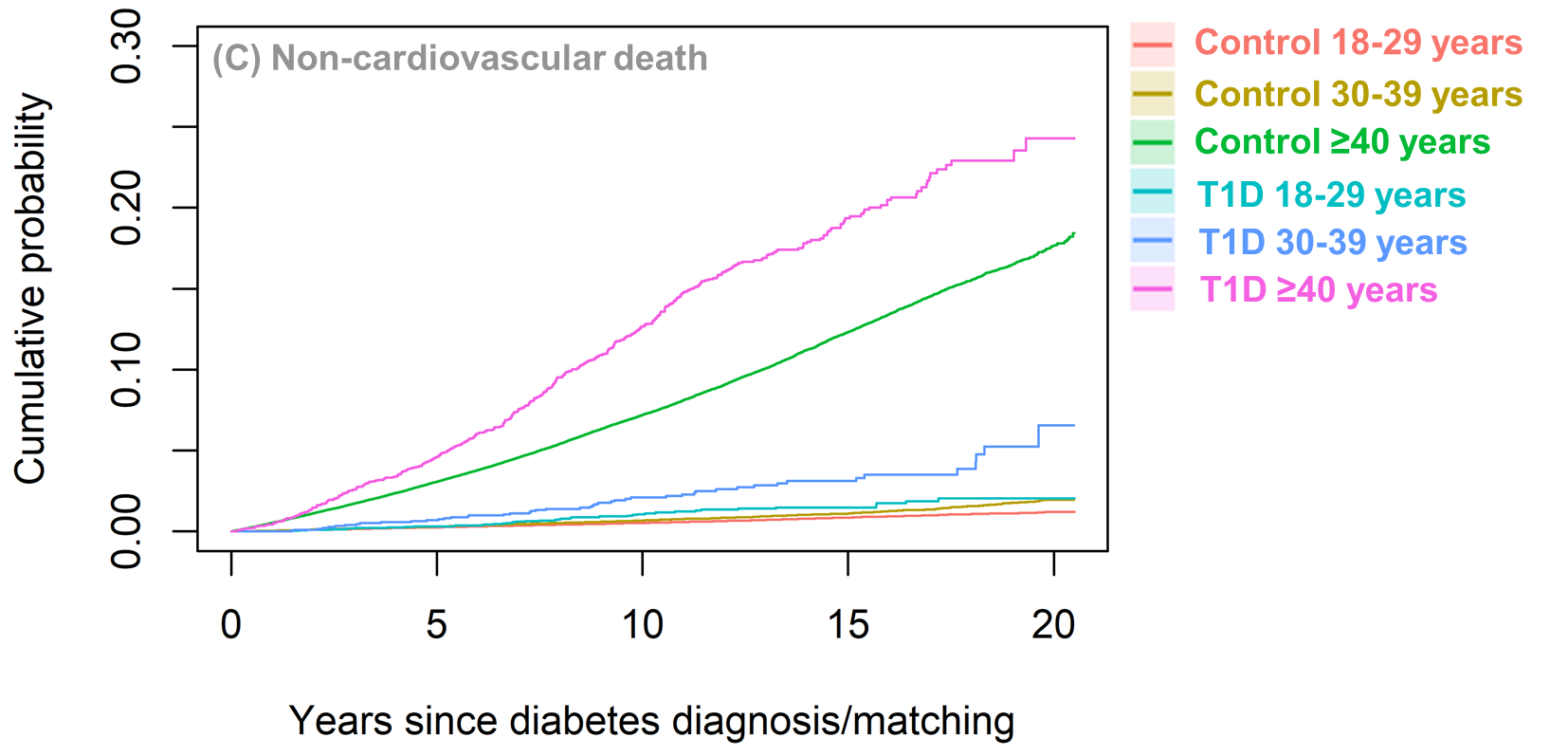


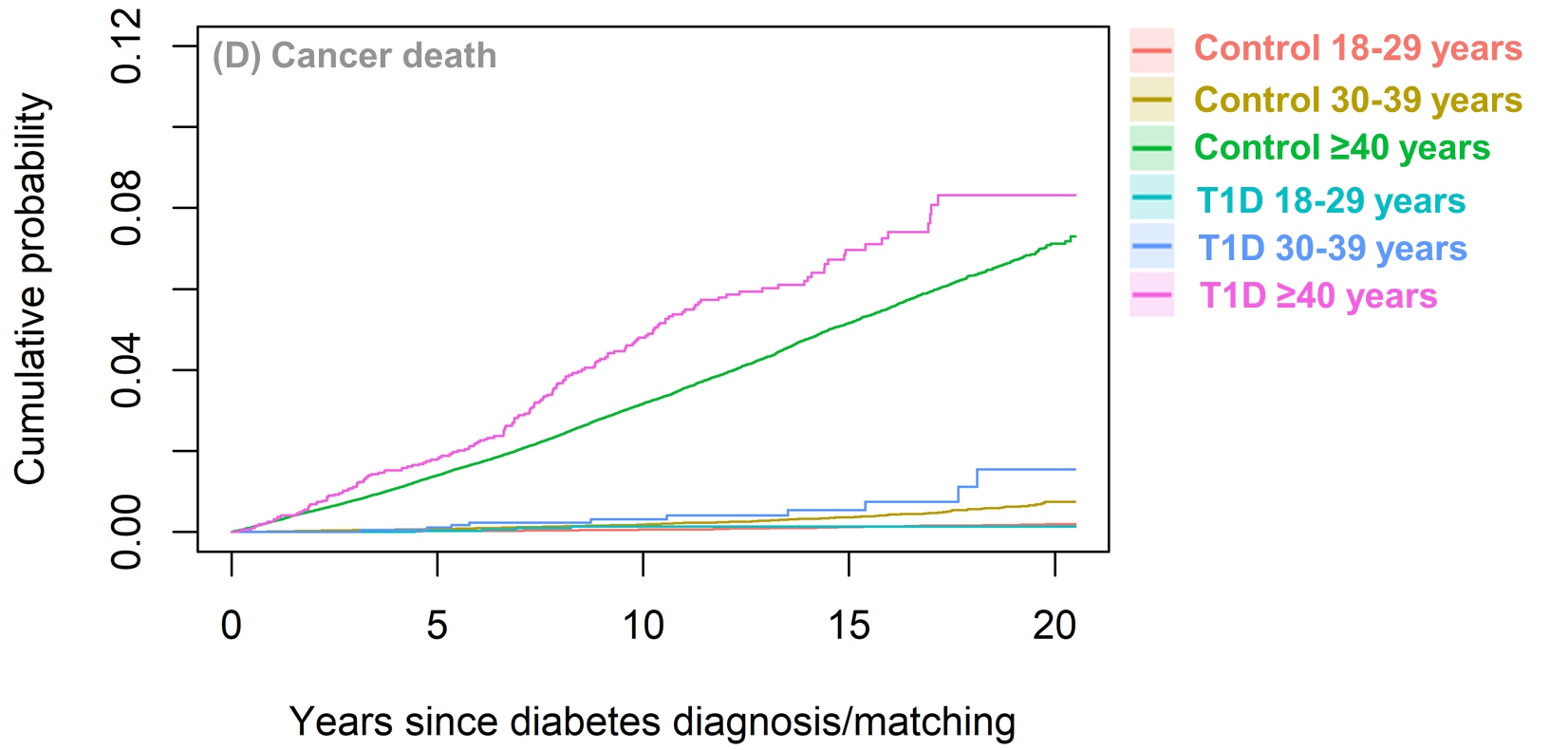


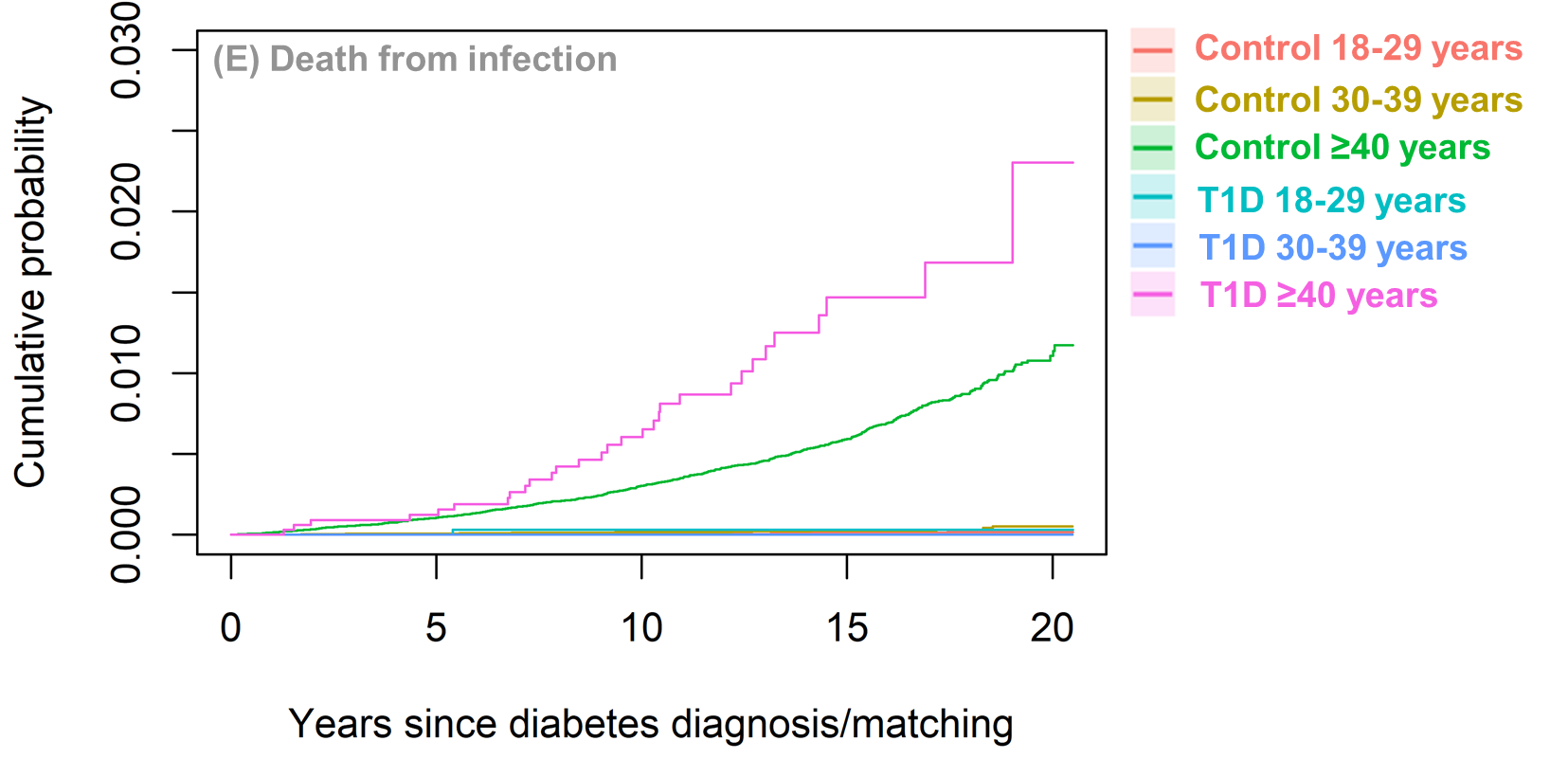


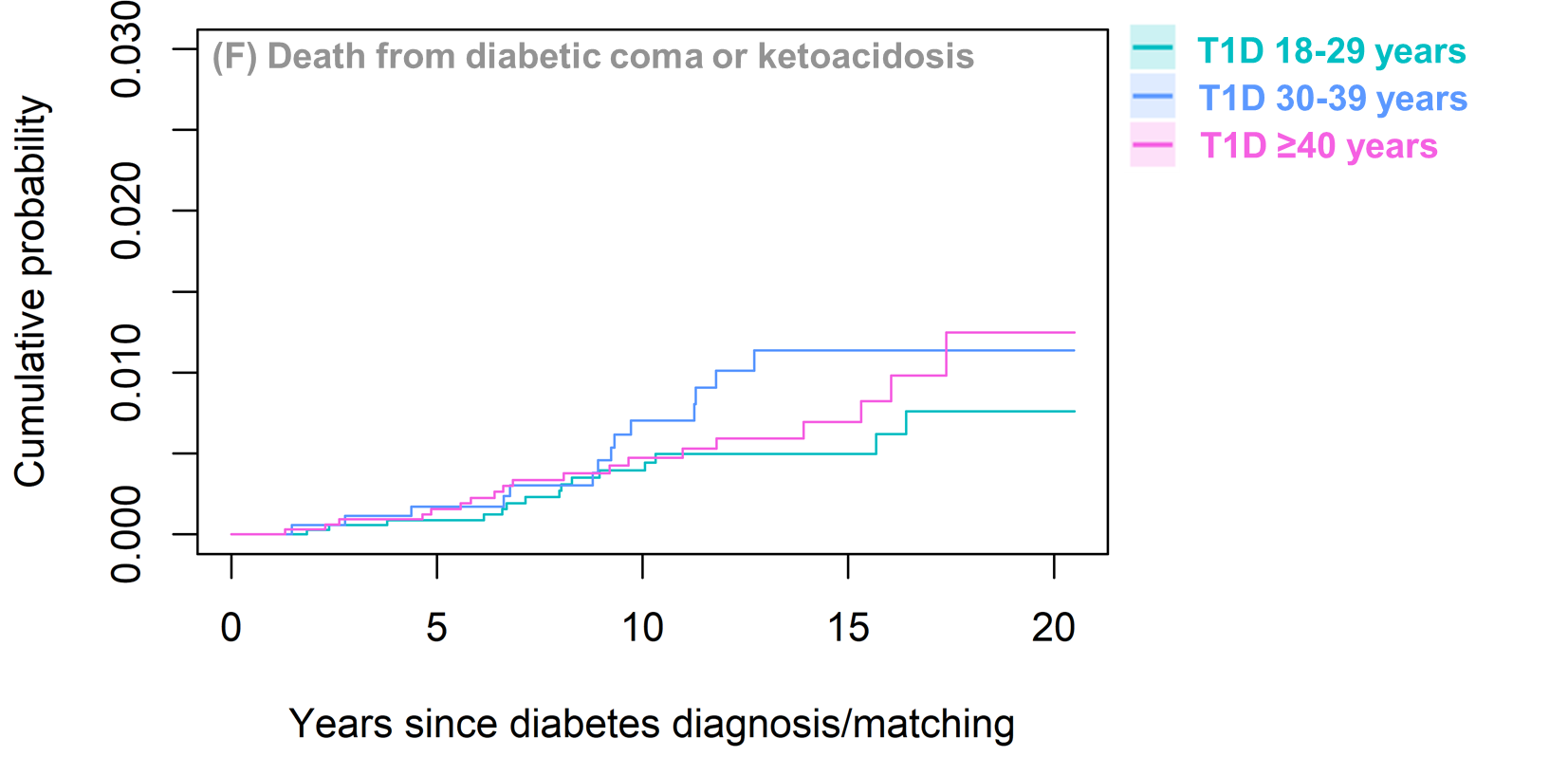


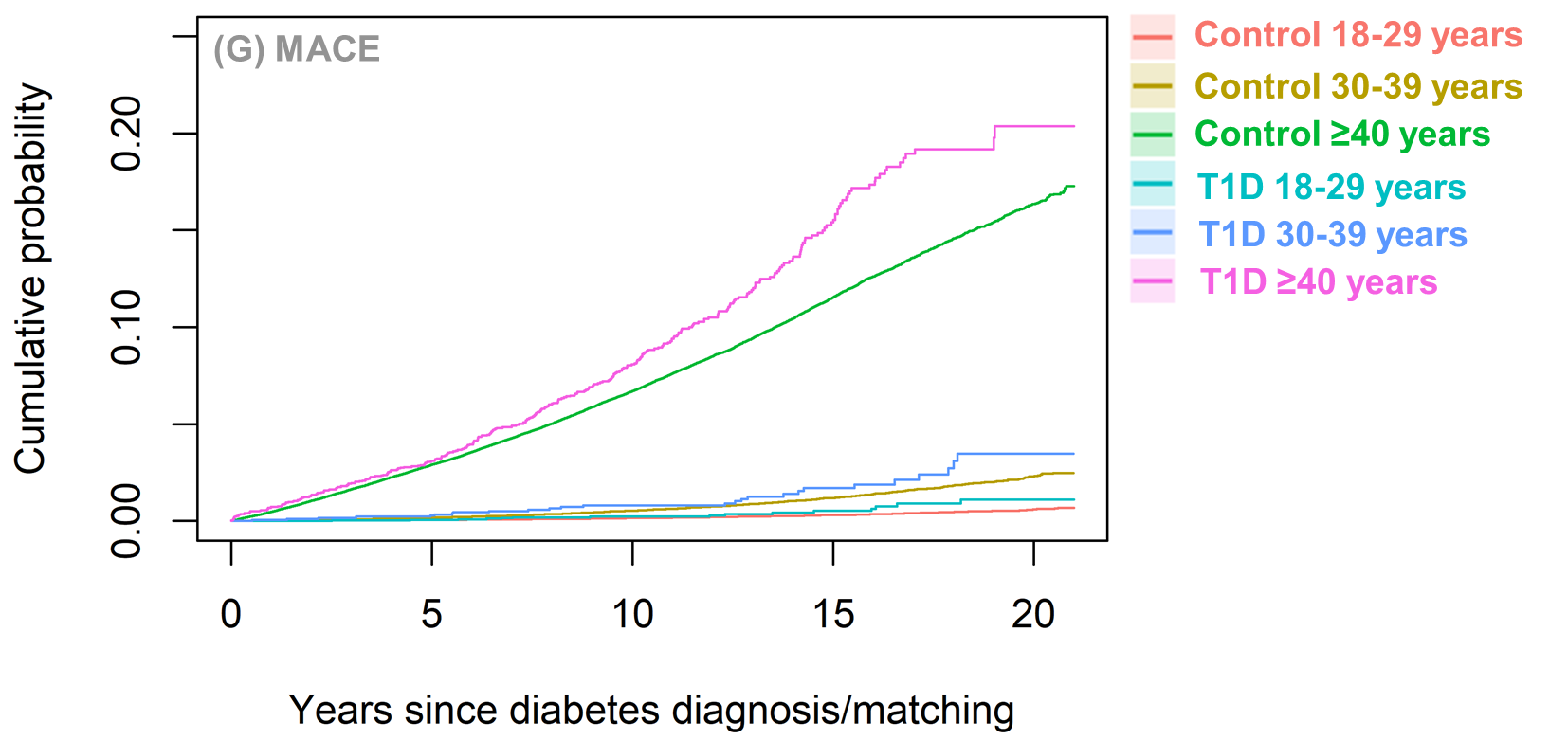


**Figure S3. Cumulative probability of different outcomes in T1D by onset-age as compared to population controls over diabetes/matching duration**

MACE: major adverse cardiovascular events; T1D: type 1 diabetes.

Cumulative probability of all-cause mortality was plotted using the Kaplan-Meier approach. Cumulative probability of cause-specific mortality and MACE was plotted using the Aalen-Johansen method to account for competing events.


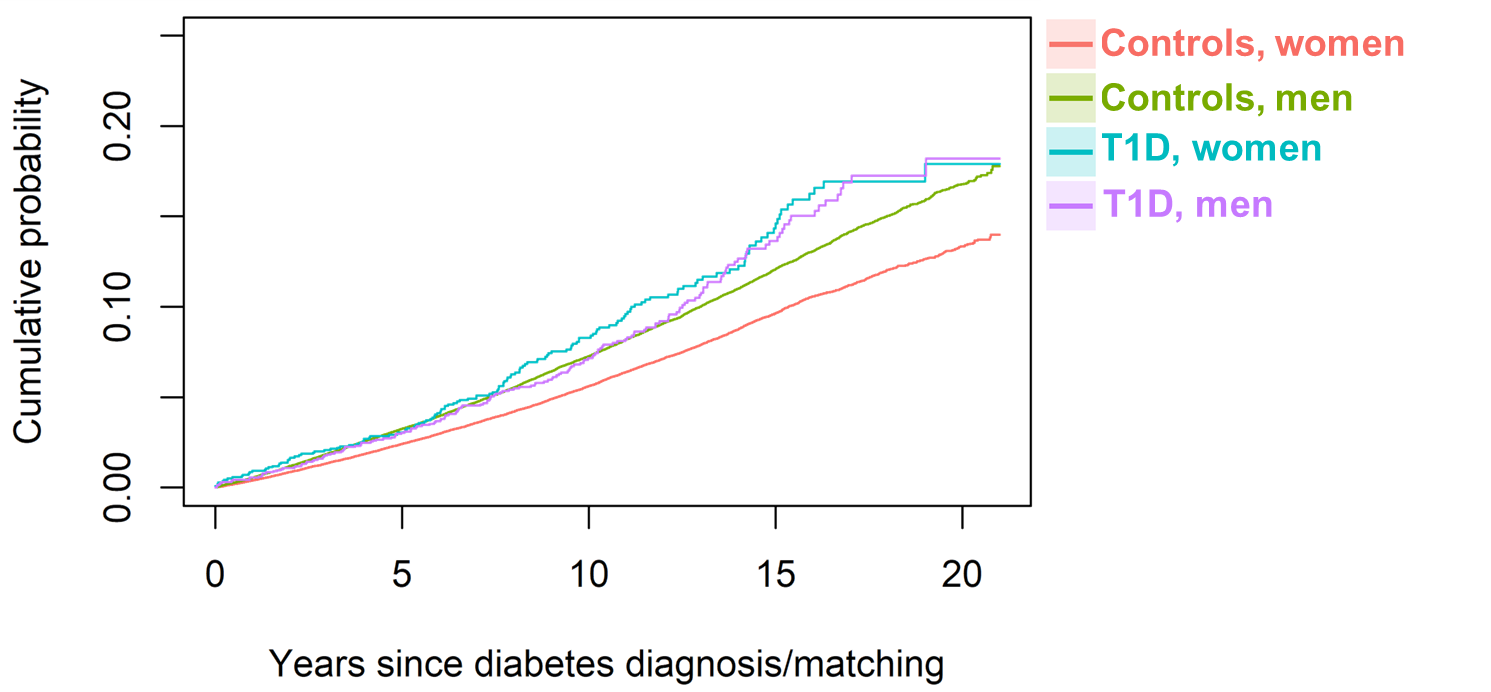


### **Figure S4. Cumulative probability of MACE in T1D diagnosed after age 40 years and matched controls by sex**

MACE: major adverse cardiovascular events; T1D: type 1 diabetes

Cumulative probability of MACE was calculated based on cumulative incidence function using the Aalen-Johansen method to account for competing events.


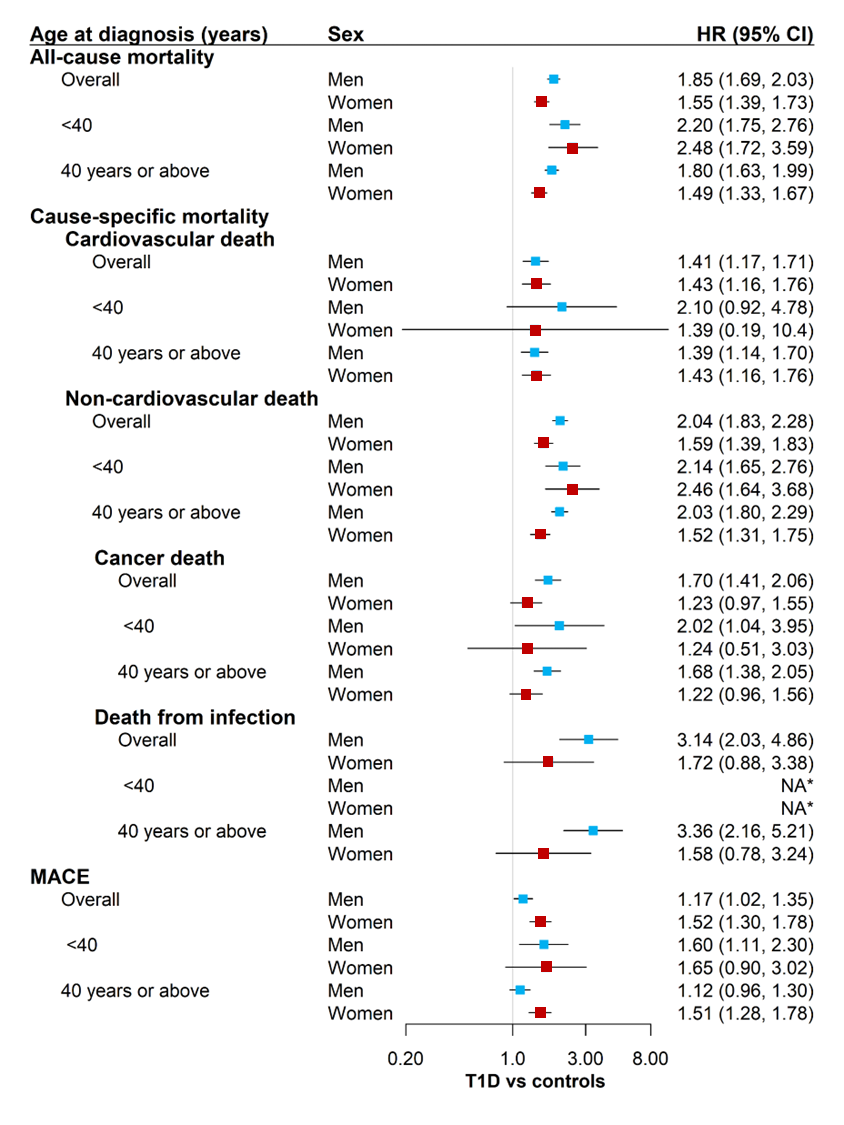


**Figure S5. Hazard ratios (95% CI) for mortality and MACE in adult-onset type 1 diabetes compared to population controls by age at diagnosis/matching and sex**

HR: hazard ratio; CI: confidence interval; MACE: major adverse cardiovascular events; T1D: type 1 diabetes.

Cox models for comparing people with T1D and population controls were fitted with diabetes/matching duration as the time scale, with adjustment for country of birth, marital status, and education, and with stratification by matching groups.

* HRs (95% CIs) were not estimated due to the small number of outcomes.


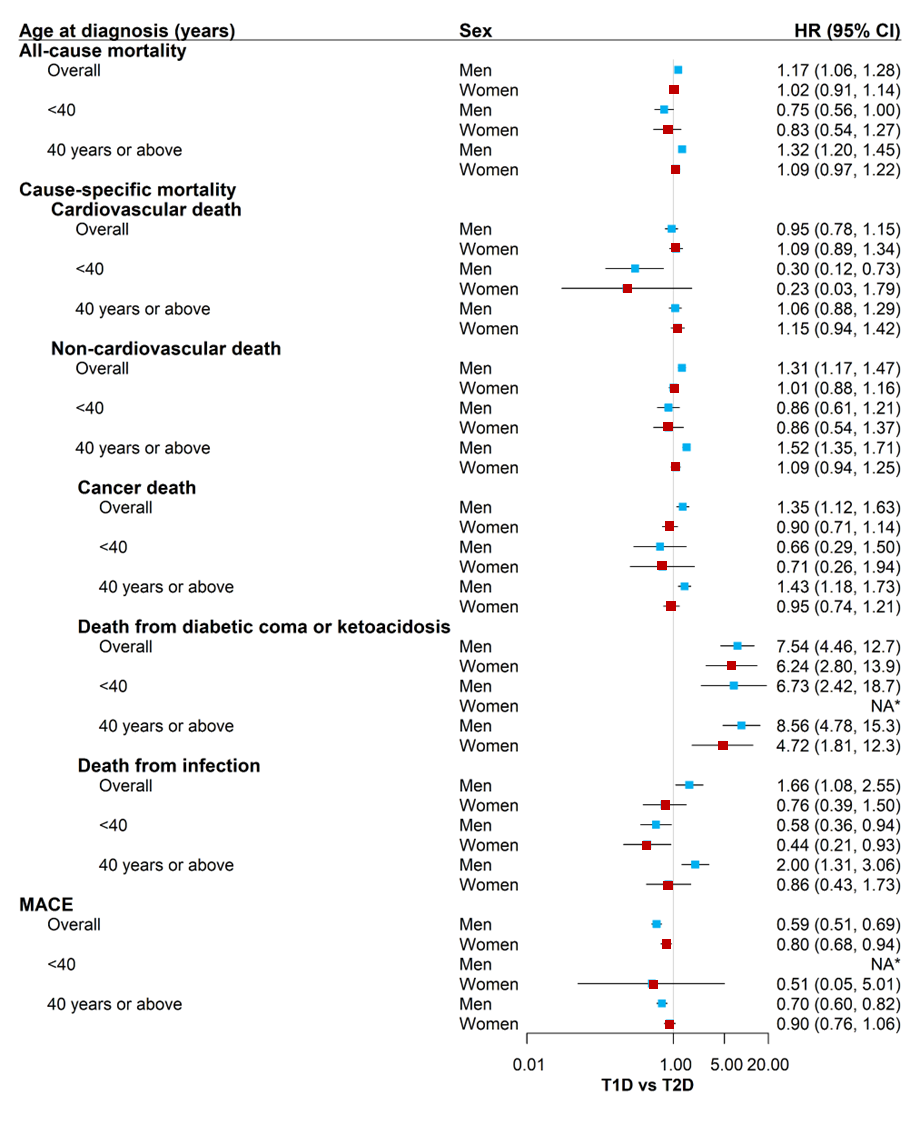


**Figure S6. Hazard ratios (95% CI) for mortality and MACE in adult-onset type 1 diabetes compared to type 2 diabetes by age at diagnosis and sex**

HR: hazard ratio; CI: confidence interval; MACE: major adverse cardiovascular events; T1D: type 1 diabetes; T2D: type 2 diabetes.

Cox models comparing people with T1D to people with T2D were fitted with attained age as the time scale, with adjustment for age and calendar year at diabetes diagnosis, country of birth, marital status, and education.

* HRs (95% CIs) were not estimated due to the small number of outcomes.


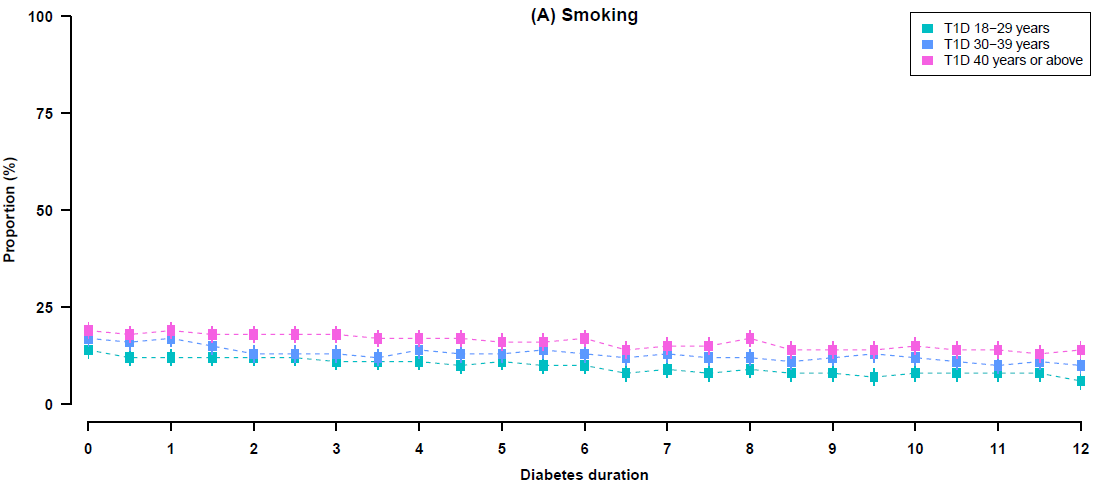


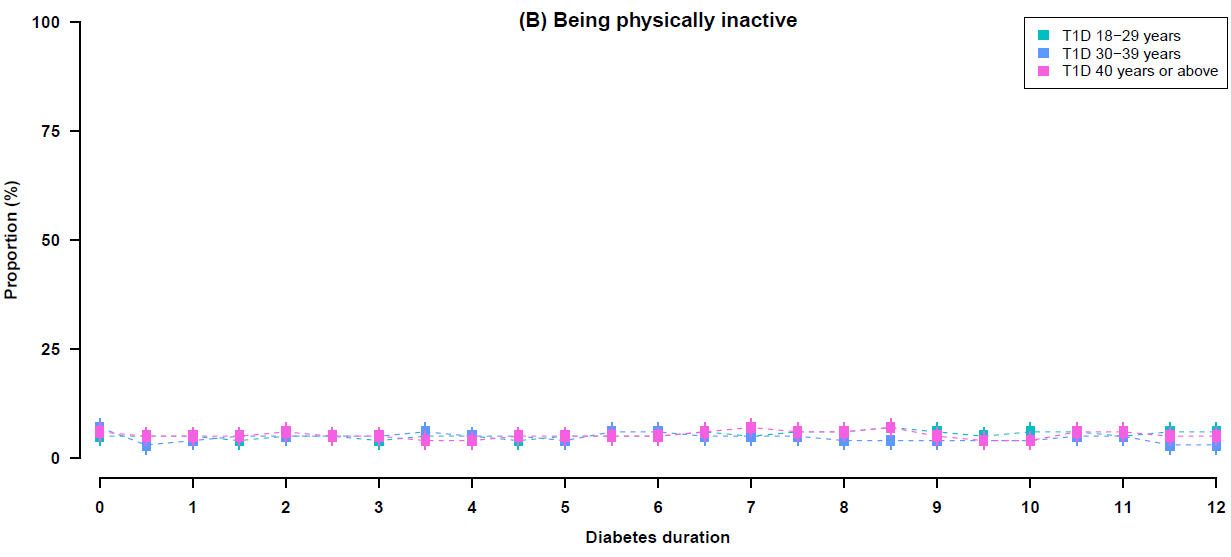


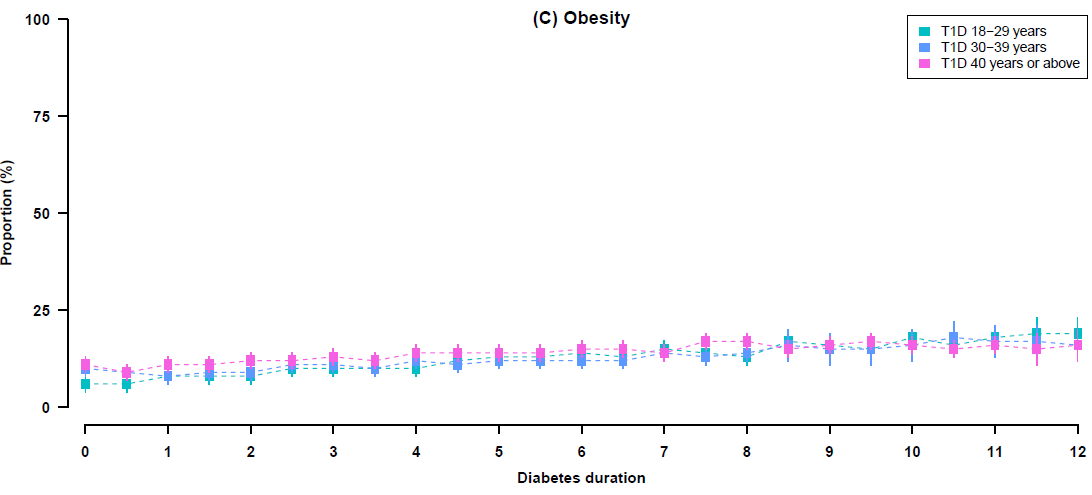


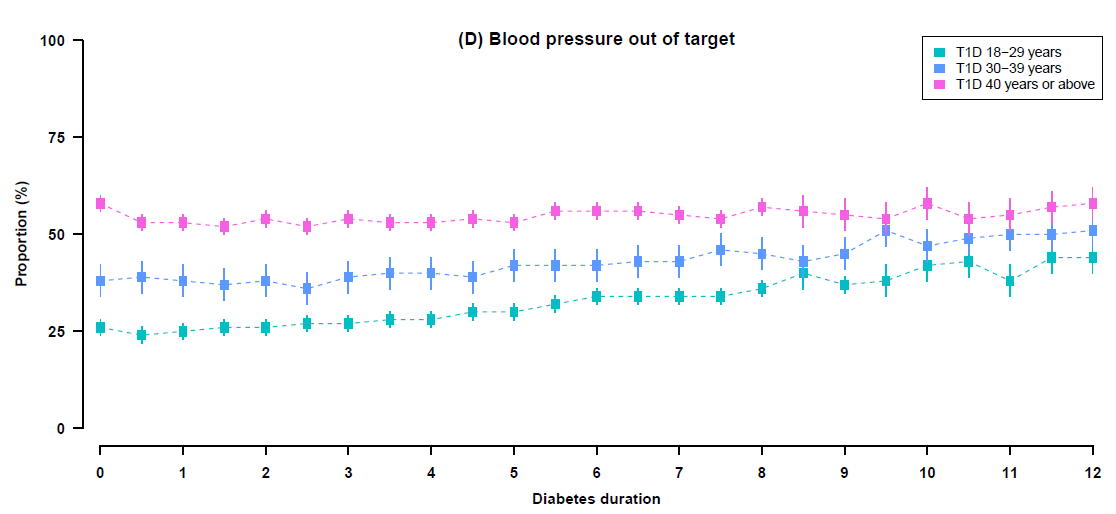


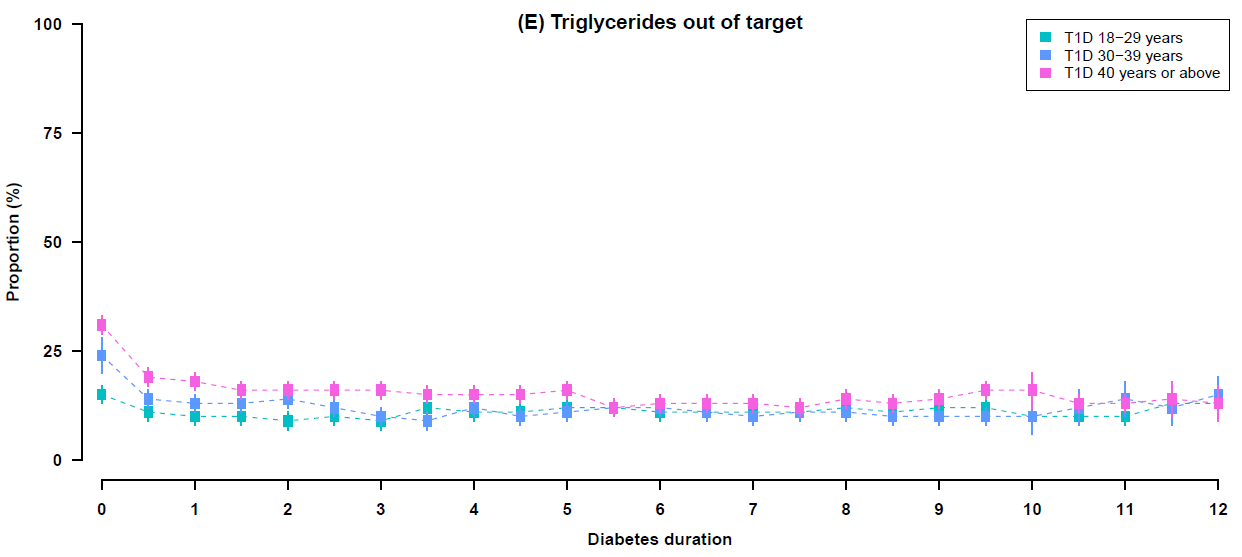


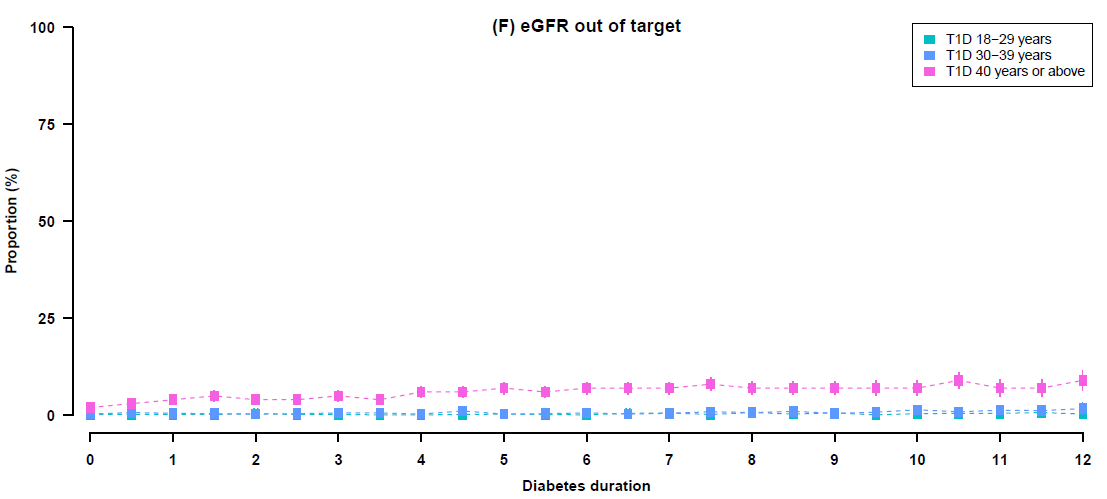


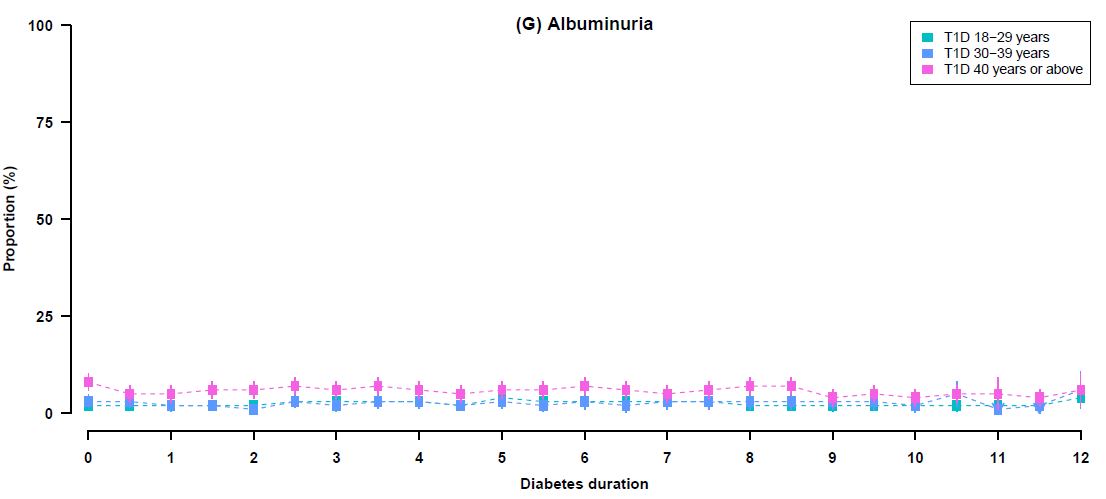


**Figure S7. Trajectories of lifestyle and clinical characteristics in people with T1D by onset-age**

T1D: type 1 diabetes; eGFR: estimated glomerular filtration rate.

The trajectory analyses were adjusted for sex and calendar year at diabetes diagnosis, with cluster-robust standard errors to account for the dependence of measures within the same individual.

Targets for different biomarkers: blood pressure <140/80 mmHg,^13^ triglycerides<1.7 mmol/L,^14^ and eGFR≥60 mL/min/1.73m^2^.

**References**

1. Eeg-Olofsson K, Cederholm J, Nilsson PM, Zethelius B, Svensson AM, Gudbjörnsdóttir S*, et al.* Glycemic control and cardiovascular disease in 7,454 patients with type 1 diabetes: an observational study from the Swedish National Diabetes Register (NDR). *Diabetes Care* 2010;**33**:1640-1646. doi: 10.2337/dc10-0398

2. Ludvigsson JF, Andersson E, Ekbom A, Feychting M, Kim JL, Reuterwall C*, et al.* External review and validation of the Swedish national inpatient register. *BMC Public Health* 2011;**11**:450. doi: 10.1186/1471-2458-11-450

3. Wettermark B, Hammar N, Fored CM, Leimanis A, Otterblad Olausson P, Bergman U*, et al.* The new Swedish Prescribed Drug Register--opportunities for pharmacoepidemiological research and experience from the first six months. *Pharmacoepidemiol Drug Saf* 2007;**16**:726-735. doi: 10.1002/pds.1294

4. Ann-Marie Svensson BE, Ebba Linder, Ia Almskog, Victoria Hermansson-Carter, Katarina Eeg-Olofsson, Mervete Miftaraj, Soffia Gudbjörnsdottir, Stefan Franzén. Nationwide results 1996-2020, Swedish National Diabetes Register NDR. In.

5. Steineck I, Cederholm J, Eliasson B, Rawshani A, Eeg-Olofsson K, Svensson AM*, et al.* Insulin pump therapy, multiple daily injections, and cardiovascular mortality in 18,168 people with type 1 diabetes: observational study. *Bmj* 2015;**350**:h3234. doi: 10.1136/bmj.h3234

6. Linn W, Persson M, Rathsman B, Ludvigsson J, Lind M, Andersson Franko M*, et al.* Estimated glucose disposal rate is associated with retinopathy and kidney disease in young people with type 1 diabetes: a nationwide observational study. *Cardiovasc Diabetol* 2023;**22**:61. doi: 10.1186/s12933-023-01791-x

7. Rawshani A, Rawshani A, Franzén S, Eliasson B, Svensson AM, Miftaraj M*, et al.* Mortality and Cardiovascular Disease in Type 1 and Type 2 Diabetes. *N Engl J Med* 2017;**376**:1407-1418. doi: 10.1056/NEJMoa1608664

8. Wang H, Cordiner RLM, Huang Y, Donnelly L, Hapca S, Collier A*, et al.* Cardiovascular Safety in Type 2 Diabetes With Sulfonylureas as Second-line Drugs: A Nationwide Population-Based Comparative Safety Study. *Diabetes Care* 2023;**46**:967-977. doi: 10.2337/dc22-1238

9. Richardson TL, Jr., Halvorson AE, Hackstadt AJ, Hung AM, Greevy R, Grijalva CG*, et al.* Primary Occurrence of Cardiovascular Events After Adding Sodium-Glucose Cotransporter-2 Inhibitors or Glucagon-like Peptide-1 Receptor Agonists Compared With Dipeptidyl Peptidase-4 Inhibitors: A Cohort Study in Veterans With Diabetes. *Ann Intern Med* 2023. doi: 10.7326/m22-2751

10. Ytterberg SR, Bhatt DL, Mikuls TR, Koch GG, Fleischmann R, Rivas JL*, et al.* Cardiovascular and Cancer Risk with Tofacitinib in Rheumatoid Arthritis. *N Engl J Med* 2022;**386**:316-326. doi: 10.1056/NEJMoa2109927

11. Bebu I, Schade D, Braffett B, Kosiborod M, Lopes-Virella M, Soliman EZ*, et al.* Risk Factors for First and Subsequent CVD Events in Type 1 Diabetes: The DCCT/EDIC Study. *Diabetes Care* 2020;**43**:867-874. doi: 10.2337/dc19-2292

12. Gordis L. More on risk: estimating the potential for prevention. In. Epidemiology. Philadelphia: Elsevier Inc; 2014, p234.

13. Rawshani A, Rawshani A, Franzén S, Sattar N, Eliasson B, Svensson AM*, et al.* Risk Factors, Mortality, and Cardiovascular Outcomes in Patients with Type 2 Diabetes. *N Engl J Med* 2018;**379**:633-644. doi: 10.1056/NEJMoa1800256

14. Standards of Medical Care in Diabetes-2022 Abridged for Primary Care Providers. *Clin Diabetes* 2022;**40**:10-38. doi: 10.2337/cd22-as01
